# Whole-transcriptome sequencing–based concomitant detection of viral and human genetic determinants of cutaneous lesions

**DOI:** 10.1172/jci.insight.156021

**Published:** 2022-04-22

**Authors:** Amir Hossein Saeidian, Leila Youssefian, Charles Y. Huang, Fahimeh Palizban, Mahtab Naji, Zahra Saffarian, Hamidreza Mahmoudi, Azadeh Goodarzi, Soheila Sotoudeh, Fatemeh Vahidnezhad, Maliheh Amani, Narjes Tavakoli, Ali Ajami, Samaneh Mozafarpoor, Mehrdad Teimoorian, Saeed Dorgaleleh, Sima Shokri, Mohammad Shenagari, Nima Abedi, Sirous Zeinali, Paolo Fortina, Vivien Béziat, Emmanuelle Jouanguy, Jean-Laurent Casanova, Jouni Uitto, Hassan Vahidnezhad

**Affiliations:** 1 Department of Dermatology and Cutaneous Biology and; 2 Jefferson Institute of Molecular Medicine, Sidney Kimmel Medical College, and; 3 Genetics, Genomics and Cancer Biology PhD program, College of Life Sciences, Thomas Jefferson University, Philadelphia, Pennsylvania, USA.; 4 Laboratory of Complex Biological Systems and Bioinformatics, Institute of Biochemistry and Biophysics, University of Tehran, Tehran, Iran.; 5 Imam Khomeini Hospital and; 6 Department of Dermatology, Razi Hospital, Tehran University of Medical Sciences, Tehran, Iran.; 7 Department of Dermatology, Rasool Akram Medical Complex Clinical Development Center, School of Medicine, Iran University of Medical Sciences, Tehran, Iran.; 8 Department of Dermatology, Children’s Medical Center, Pediatric Center of Excellence, Tehran University of Medical Sciences, Tehran, Iran.; 9 UCSC Silicon Valley Extension, University of California, Santa Cruz, Santa Clara, California, USA.; 10 Department of Dermatology, Gonabad University of Medical Sciences, Gonabad, Iran.; 11 Nobel Laboratory, Isfahan, Iran.; 12 Department of Dermatology, Skin Disease and Leishmaniasis Research Center, Isfahan University of Medical Sciences, Isfahan, Iran.; 13 Stem Cell Research Center and; 14 Student Research Committee, Golestan University of Medical Sciences, Gorgan, Iran.; 15 Department of Allergy and Clinical Immunology, Rasool Akram Hospital, Iran University of Medical Sciences, Tehran, Iran.; 16 Department of Microbiology, School of Medicine, Gastrointestinal and Liver Disease Research Center, Guilan University of Medical Sciences, Rasht, Iran.; 17 Depatment of Bioinformatics, Institute of Biochemistry and Biophysics, University of Tehran, Tehran, Iran.; 18 Kawsar Human Genetics Research Center, Tehran, Iran.; 19 Department of Cancer Biology, Sidney Kimmel Cancer Center, Thomas Jefferson University, Philadelphia, Pennsylvania, USA.; 20 Department of Translational and Precision Medicine, Sapienza University, Rome, Italy.; 21 St. Giles Laboratory of Human Genetics of Infectious Diseases, Rockefeller Branch, Rockefeller University, New York, New York, USA.; 22 Laboratory of Human Genetics of Infectious Diseases, Necker Branch, INSERM U1163, Necker Hospital for Sick Children, Paris, France.; 23 Imagine Institut, Paris University, Paris, France.; 24 Howard Hughes Medical Institute, New York, New York, USA.; 25 Department of Pediatrics, Necker Hospital for Sick Children, Paris, France.

**Keywords:** Genetics, Infectious disease, Bioinformatics, Genetic diseases, Molecular diagnosis

## Abstract

Severe viral infections of the skin can occur in patients with inborn errors of immunity (IEI). We report an all-in-one whole-transcriptome sequencing–based method by RNA-Seq on a single skin biopsy for concomitantly identifying the cutaneous virome and the underlying IEI. Skin biopsies were obtained from healthy and lesional skin from patients with cutaneous infections suspected to be of viral origin. RNA-Seq was utilized as the first-tier strategy for unbiased human genome-wide rare variant detection. Reads unaligned to the human genome were utilized for the exploration of 926 viruses in a viral genome catalog. In 9 families studied, the patients carried pathogenic variants in 6 human IEI genes, including *IL2RG*, *WAS*, *CIB1*, *STK4*, *GATA2*, and *DOCK8*. Gene expression profiling also confirmed pathogenicity of the human variants and permitted genome-wide homozygosity mapping, which assisted in identification of candidate genes in consanguineous families. This automated, online, all-in-one computational pipeline, called VirPy, enables simultaneous detection of the viral triggers and the human genetic variants underlying skin lesions in patients with suspected IEI and viral dermatosis.

## Graphical abstract

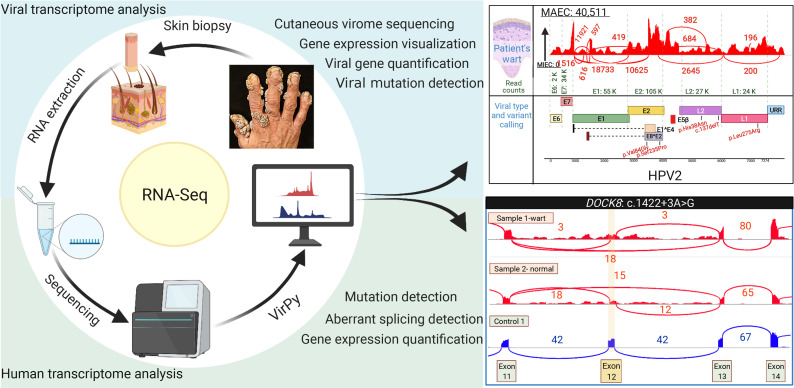

## Introduction

Cutaneous viral infections are common and phenotypically manifest from localized to generalized lesions (1). In dermatological settings, patients are usually examined for the clinical management of their skin manifestations, including virological identification, rarely accompanied by an investigation of the genetic susceptibility to the infections. Besides, pathogenic viruses are numerous, and unrelated viruses may cause similar clinical manifestations (2). Overall, these confounding factors necessitate the development of rapid, accurate, and cost-effective laboratory methods to enhance the effective diagnostics and case management of patients with suspected viral infections and the implementation of mitigation strategies for viral transmission in communities.

Many viral infections are self-limiting or asymptomatic in immunocompetent individuals without requiring medical intervention (3). In contrast, the same viral agents can cause devastating disease in patients with inborn errors of immunity (IEI) due to single-gene mutations (4). Known IEI comprise individually rare but collectively diverse genetic defects, with as many as 430 mutated genes (5, 6), which impair the development or function, or both, of cells involved in immunity to infection. Papillomaviruses, with almost 450 distinct HPV types (7), are an ancient group of DNA viruses that infect the mucocutaneous tissue of their targets, including *Homo sapiens*. However, as mentioned above, most HPVs cause infections that are innocuous and asymptomatic unless the host’s immune system is compromised.

With next-generation sequencing (NGS) techniques, we considered the possibility of identifying the human genetic susceptibility and skin virome repertoire efficiently and accurately from whole-transcriptome sequencing (RNA-Seq). A few previous studies aimed to investigate the virome from RNA-Seq data, using pipelines developed for virus detection only (8–11). However, there is a need for a computational pipeline for simultaneous detection of viral skin infections and underlying genetic mutations in the host. This study aimed to fill this gap of technological knowledge.

## Results

### VirPy, an all-in-one RNA-Seq–based tool.

**Figure 1:**
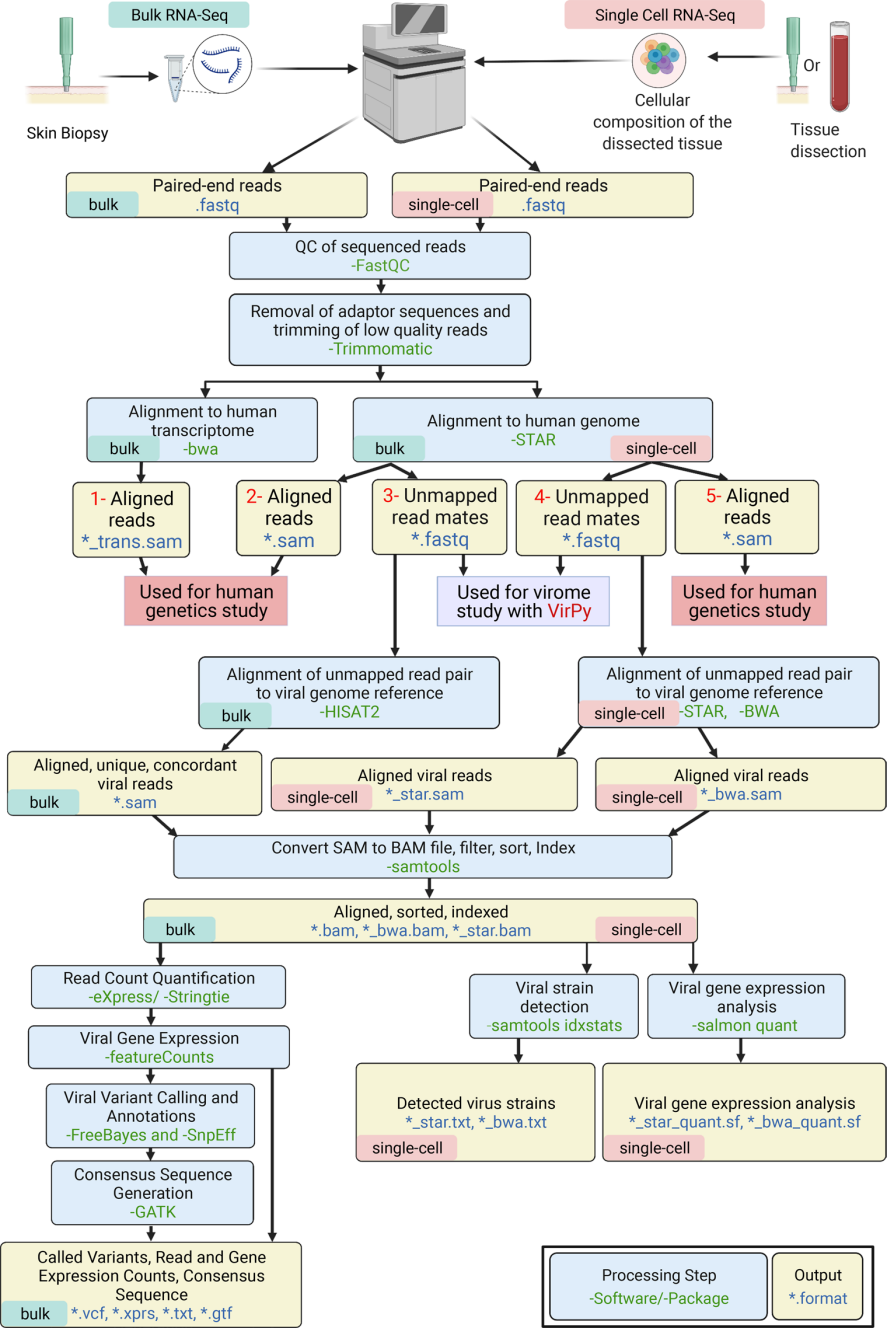
Schematic overview of the proposed VirPy pipeline. A comprehensive pipeline of RNA-Seq data for concomitant virome detection and host variant calling based on both bulk and single-cell sequencing methods. The figure was generated in BioRender.

The VirPy pipeline performs a multitude of computational procedures to generate a complete, comprehensive analysis of both the patient’s genetic mutation and virome. The pipeline was developed using the Python programming language employing third-party tools, including STAR (12), HISAT2 (13), SAMtools (14), eXpress (15), Subread featureCounts (16), FreeBayes (http://arxiv.org/abs/1207.3907), Salmon (17), and SnpEff packages (18). RNA extraction and whole-transcriptome sequencing were performed as previously described (19). In this process, the quality of RNA-Seq data is assessed by FastQC, and according to the results, trimming is considered. The high-quality reads in the FASTQ files are used as inputs for the pipeline. FASTQ paired-end reads are aligned to the human genome reference (GRCh37) using the STAR RNA-Seq aligner. Unaligned reads or reads only partially aligned to the human genome are assumed to be nonhuman in origin and represent genetic material from microorganism species. The nonhuman paired mates are extracted and realigned using the HISAT2 aligner for bulk RNA-Seq and STAR/BWA mem for single-cell RNA-Seq (scRNA-Seq) to a compiled viral genome reference containing 926 unique viral species obtained from the National Center for Biotechnology Information (NCBI), including over 400 known types of HPV (7). The precompiled viral genome reference database can be updated and modified with new entries for future usage, allowing for flexibility depending on the types of analyses or viral species of interest. The virus-containing SAM file is filtered to contain only concordant pairs. Reads aligned to the viral genome reference are formatted into the aligned, sorted, and indexed BAM files, which can be visualized in Integrated Genome Viewer (IGV) for bulk RNA-Seq (Figure 1 and ref. 20).

A series of methodological tasks were performed to analyze the viral genetic material. In bulk RNA-Seq the relative abundance of sequences was quantified by eXpress, which analyzes the unique abundance of reads for each virus present in the sample, and reports both the total and unique read counts per virus. An empirical cutoff of the number of unique reads mapped to a virus genome was applied to filter out artifacts of sampling, alignment, and noise that do not represent true viral sequences. In addition, viruses commonly detected in healthy human tissue samples present in low, residual quantities (i.e., murine leukemia virus and/or human herpesvirus 5 and 6) were excluded from the final list of detected viruses. The default cutoff was 50 “unique reads” that are concordantly aligned exactly 1 time and without secondary alignments. Reads that did not align concordantly or had additional secondary alignments were filtered out of the BAM file to avoid incorrectly attributing genome segments that were common between viruses. In scRNA-Seq, the viral copy number was measured by using SAMtools idxstats, and the read counts were quantified at the individual viral gene level by Salmon. Next steps for bulk RNA-Seq included a) quantification of the absolute count of viral features (i.e., exons and transcripts) by the featureCounts tool in the Subread package, b) viral variant analysis by the variant caller FreeBayes, and c) annotation of the output variant call file with SnpEff. A consensus sequence is then generated from the variant call file using GATK (21) and Picard tools (https://broadinstitute.github.io/picard/).

We integrated the previously developed pipeline of whole-transcriptome analysis for pathogenic sequence variants by RNA-Seq with our virome detection pipeline into a single-run experiment (9). In our previously published pipeline, we used genome and transcriptome references concurrently for alignment mapping to identify genomic mutations with an overall yield of over 85%. The details of the detection, quantification, and variant calling for the human transcriptome within the VirPy module are described elsewhere (19, 22). We have now expanded the VirPy tool for simultaneous detection of both virome repertoire and the underlying causal mutations in the host genome from bulk and scRNA-Seq FASTQ files. The VirPy workflow was developed using the Python programming language employing third-party tools and is publicly available for free use at https://github.com/Vahidnezhad-Lab/VirPy (commit ID 8e67ee7244e23d21a198180206a5d9dee2570b5f). We have also deployed a publicly available web-based application for VirPy, available at https://www.VirPy.org

### Validation and utility of VirPy.

In the current study, all data sets (in-house and public) were examined experimentally, and the viral infection was confirmed with other standard methods such as reverse transcription PCR, real-time PCR, and histopathology (i.e., presence of koilocytes). Our results demonstrated congruence between the viruses detected by the proposed pipeline and the experimentally confirmed serotypes. For instance, using VirPy, we reanalyzed the HPV repertoire in 30 head and neck (H&N) cancer samples (15 samples, before and after treatment). The results from our transcriptome analysis were in line with the L1 staining and real-time PCR results that were recently published (23). Using VirPy, we were able to detect different HPVs in H&N cancer samples, including HPV16 in 21 samples, HPV18 in 1 sample, and HPV33 in 2 samples.

**Figure 2:**
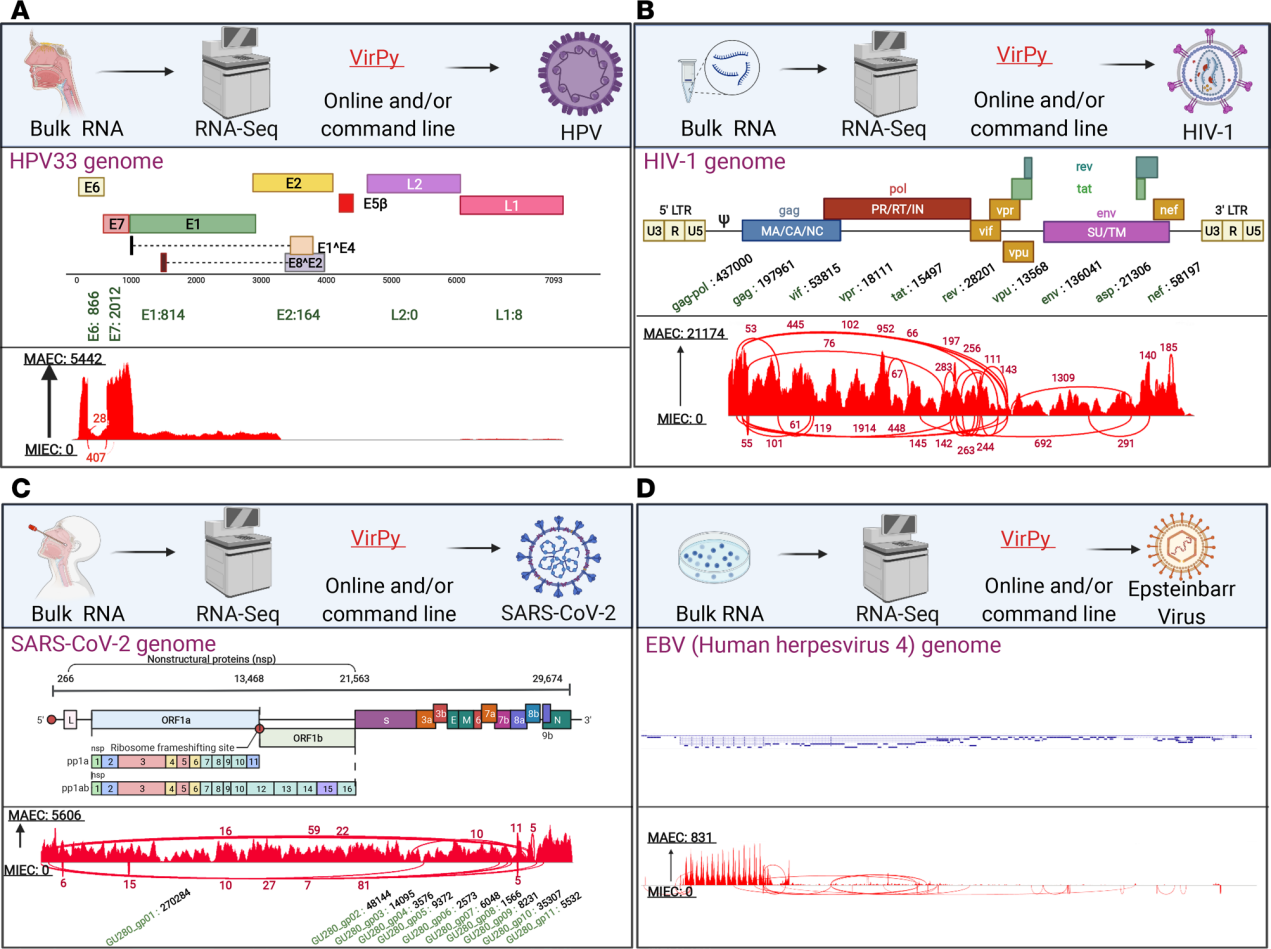
Validation of the proposed pipeline on in-house and publicly available bulk RNA-Seq data. Pipeline results from HPV33- (**A**), HIV-1– (**B**), SARS-CoV-2– (**C**), and EBV- (**D**) infected samples on bulk RNA-Seq data. Sashimi plots showed the identified viral genes. The bar plots on the right panels illustrate the detected viruses from scRNA-Seq data. The figure was generated in BioRender. LTR, long terminal repeat; MIEC, minimum exon coverage.

**Figure 3:**
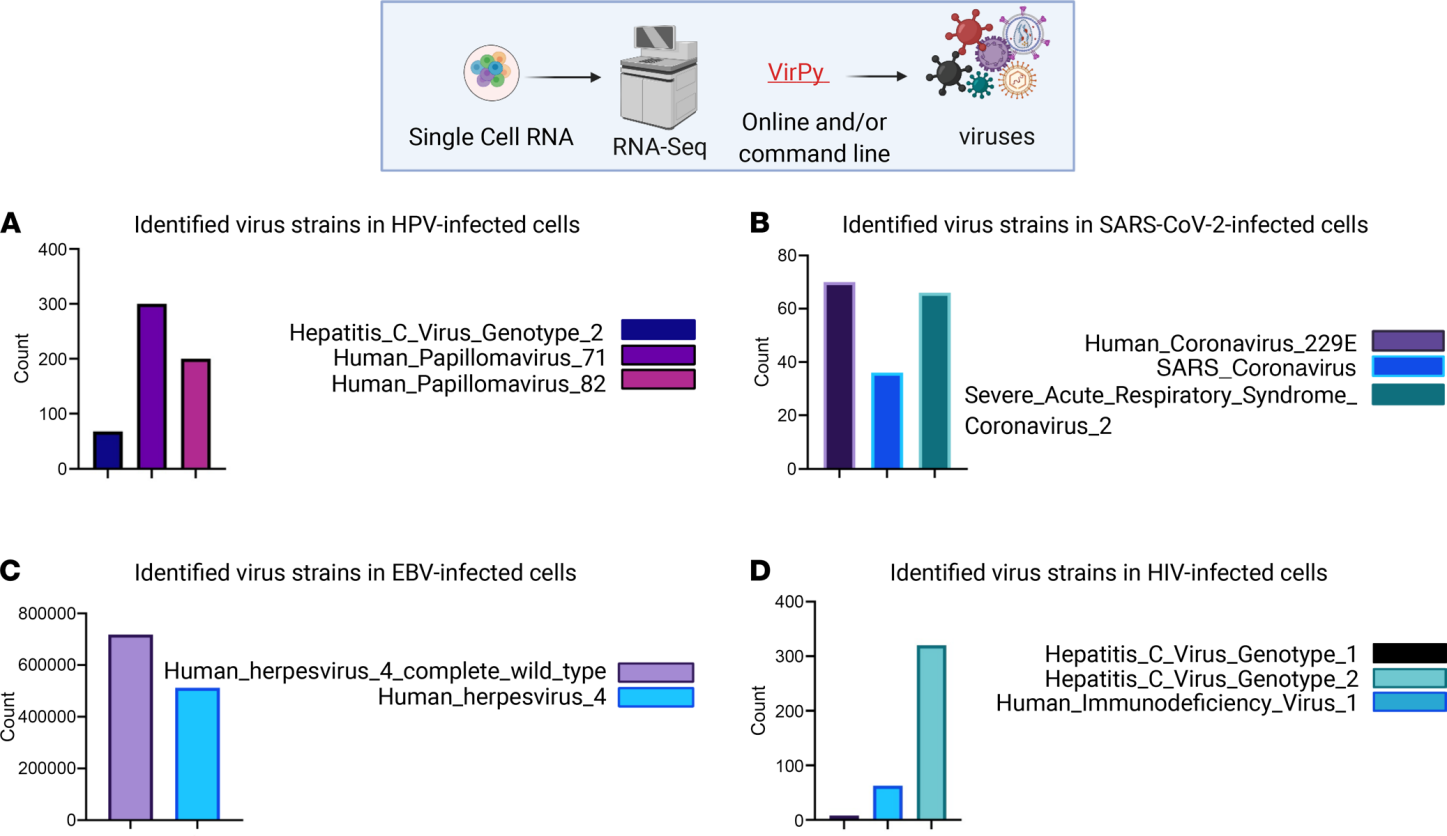
Validation of the proposed pipeline on publicly available scRNA-Seq data. Pipeline results from HPV- (**A**), SARS-CoV-2– (**B**), EBV- (**C**), and HIV-1– (**D**) infected samples on single-cell RNA-Seq data. The bar plots illustrate the detected viruses from single-cell RNA-Seq data. The figure was generated in BioRender.

The VirPy tool was also validated using publicly available bulk (Figure 2) and single-cell (Figure 3) RNA-Seq data with EBV, SARS-CoV-2, and HIV infections. After validation, we evaluated the performance of our new VirPy pipeline on patients with IEI with cutaneous viral infections, particularly HPVs. In this study, we recruited 9 distinct families including 10 patients with cutaneous viral infections who were diagnosed with IEI of unknown genetic and virome etiology. In addition, we investigated the possibility of simultaneous detection of single-gene susceptibilities and virome in patients with various viral infections, including HPV, HIV, EBV, CMV, and human T-lymphotropic virus type 1, by applying VirPy on bulk RNA-Seq skin biopsy data from 10 patients as explained in detail below. Elaborative case reports of all 9 families are available in Supplemental Tables 1 and 2 and the Supplemental Methods section.

### HPV detection in patients with IEI affected by epidermodysplasia verruciformis.

**Figure 4:**
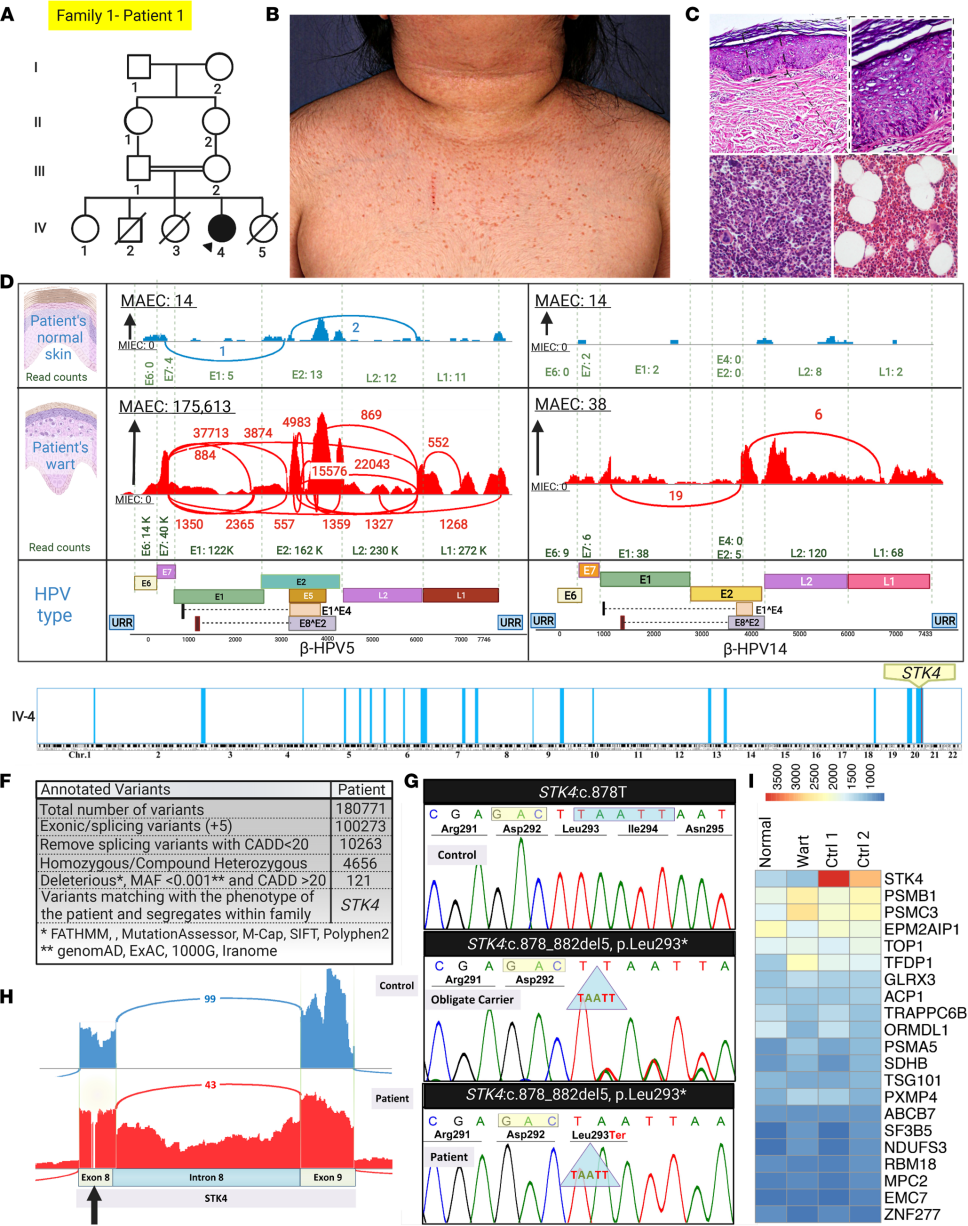
Concomitant detection of the causal single-gene defect and skin virome in a patient with IEI and EV. (**A**) Family pedigree of an 8-year-old patient initially diagnosed with EV (indicated by the arrowhead). (**B**) Widespread flat warts on the trunk. (**C**) (Upper panel) Histopathology of a flat wart on acral skin showed epidermal thickening with hyperkeratosis, acanthosis, hypergranulosis, and the presence of koilocytes with the large cytoplasmic halo. Original magnification, 10× and 40×. (Lower panel) Bone marrow histopathology at the age of 6 years showed hypercellularity with mild dysmyelopoiesis and megaloblastic changes (lower left panel), which progressively became hypocellular (cell/fat ratio 50:50) during the subsequent 3 years (right panel). (**D**) RNA-Seq analysis by VirPy detected HPV5 and HPV14 in wart samples but also insignificant levels in the patient’s normal skin. (**E**) HM performed on RNA-Seq data identified several regions of homozygosity (ROHs) (blue vertical blocks). The *STK4* gene was present within an ROH in chromosome 20. (**F**) Variants called from RNA-Seq were filtered by the indicated steps, which identified *STK4* as the pathogenic candidate gene. (**G**) Sanger sequencing of genomic DNA confirmed the presence of a homozygous mutation *STK4*: c.878_882del5 in the proband, and the parents were heterozygous carriers. (**H**) Sashimi plots showed that the mutation in *STK4* resulted in aberrant mRNA splicing, including retention of intron 8 sequences. Note the position of the mutation in the middle of exon 8 (arrow). (**I**) Heatmap visualization of transcriptome analysis revealed *STK4* was highly downregulated in comparison with the randomly selected housekeeping genes. The figure was generated in BioRender. CADD, combined annotation dependent depletion; MAF, minor allele frequency.

Epidermodysplasia verruciformis (EV) is an autosomal recessive disorder characterized by widespread flat warts, associated with the propensity to develop squamous cell carcinomas (24). EV can manifest exclusively with skin lesions (typical; ref. 25) or be syndromic (atypical; refs. 26, 27). We studied 4 patients representing 4 distinct families with the initial diagnosis of EV based on histopathology and the presence of extensive recalcitrant warts. The first case, family 1, patient 1, an 8-year-old girl born to healthy consanguineous first cousin parents, presented with erythematous papules on her neck, which eventually spread to her forehead, chest, antecubital fossa, and bilateral dorsum of the hands (Figure 4, A and B). Skin histopathology was consistent with viral infection (Figure 4C, upper panels). RNA-Seq was performed to study the underlying genetic cause and virome. HPVs 5 and 14 were detected in warts, HPV5 being clearly the predominant type as judged by the maximum exon coverage (MAEC) (Figure 4D). Insignificant amounts of these viruses were present in the normal appearing skin. The patient was suspected to have IEI, and her bone marrow biopsy revealed initial hypercellularity, which progressively developed into hypocellularity (Figure 4C, lower panels). Homozygosity mapping (HM), along with stepwise filtration of annotated variants called from RNA-Seq data, led us to characterize a potentially previously undescribed frameshift *STK4* mutation (Figure 4, E–G). Sashimi plot showed intron retention, and expression study of STK4 showed a significant reduction at the mRNA level (Figure 4, H and I). The effect of *STK4* variants on alterations of splicing regulatory element or exonic splicing enhancer (ESE) sequences were predicted using 2 independent in silico approaches: ESEfinder (http://exon.cshl.edu/ESE/) (28) and Human Splicing Finder (HSF) (http://umd.be/Redirect.html) (29). In particular, the latter tool is able to provide scores for a number of dedicated ESE motifs. Using these tools, we obtained the following result for this exonic mutation, *STK4*: c.878_882del, which is congruent with our observations of aberrant splicing shown by IGV. Due to this mutation, we have a potential alteration of splicing including activation of cryptic acceptor and donor splice sites.

**Figure 5:**
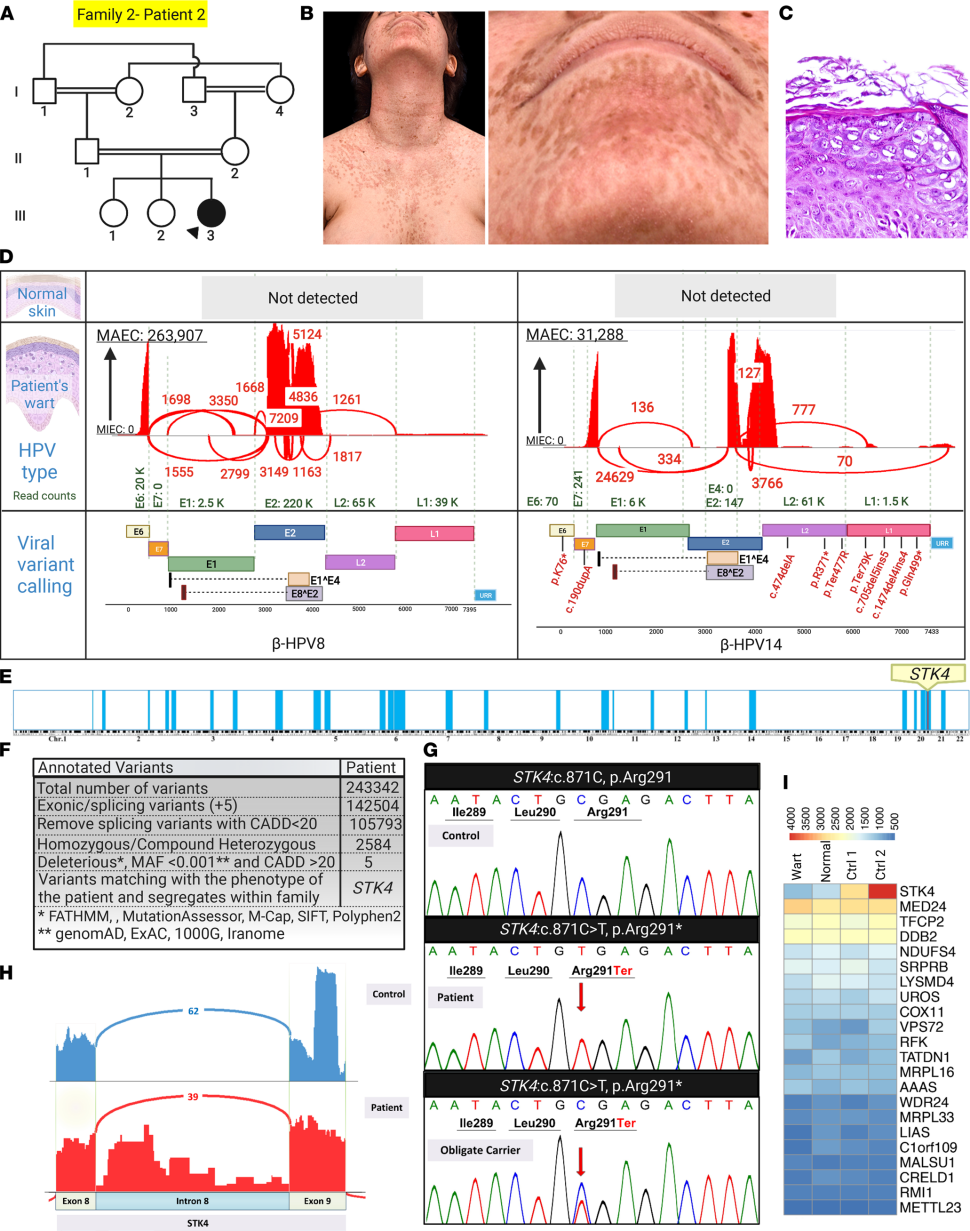
Concomitant detection of the causal single-gene defect and skin virome in a patient with IEI and EV. (**A**) Family pedigree of an 18-year-old patient (indicated by the arrowhead) with EV shows extensive consanguinity. (**B**) Flat warts on the face and trunk of the proband. (**C**) Histopathology of a flat wart on acral skin showed epidermal thickening with hyperkeratosis, acanthosis, hypergranulosis, and the presence of koilocytes with large cytoplasmic halos. (**D**) RNA-Seq analysis by VirPy detected HPV8 and HPV14 in wart samples but not in the patient’s normal tissue skin. Sequence variants identified in HPV14 are indicated in red. (**E**) HM performed on RNA-Seq data identified several ROHs (blue vertical blocks) in the patient. The *STK4* variants aligned within an ROH in chromosome 20. (**F**) Variants called from RNA-Seq were filtered by the indicated steps and identified *STK4* as the pathogenic candidate gene. (**G**) Sanger sequencing of genomic DNA confirmed the presence of a homozygous mutation in the proband, and the parents were heterozygous. (**H**) Sashimi plot showed that the mutation in *STK4* resulted in aberrant mRNA splicing, including retention of intron 8 sequences. (**I**) Heatmap visualization of transcriptome analysis revealed *STK4* as the most downregulated gene among the randomly selected housekeeping genes when compared with controls. The figure was generated in BioRender.

The second case, family 2, patient 2, is an 18-year-old woman born to healthy double first cousin consanguineous parents (Figure 5A). She initially presented with a history of frequent, prolonged bacterial/viral upper and lower respiratory tract infections during infancy. At 4 years of age, pale/erythematous nonpruritic macules and papules appeared initially on the neck and later spread to the chest, face, and upper extremities; these lesions are still present at the age of 18 years, and histopathology was consistent with a viral infection (Figure 5, B and C). HPVs 8 and 14 were detected in warts, but not in normal appearing skin, and HPV8 was the predominant type (Figure 5D). Variant calling from RNA-Seq data, homozygosity mapping, and stepwise bioinformatics analysis revealed a potentially previously undescribed nonsense *STK4*: c.871C>T, p.Arg291* mutation (Figure 5, E–G). Sashimi plot revealed retention of intron 8 sequences, and the corresponding mRNA level was significantly reduced when compared with the controls (Figure 5, H and I). Using HSF, the effect of this nonsense variant was examined, and significant alteration of auxiliary sequences including ESE/exonic splicing silencer (ESS) motif ratio of –6 was predicted. Also due to this mutation, potential alteration of splicing, such as activation of a cryptic donor site, was predicted.

**Figure 6:**
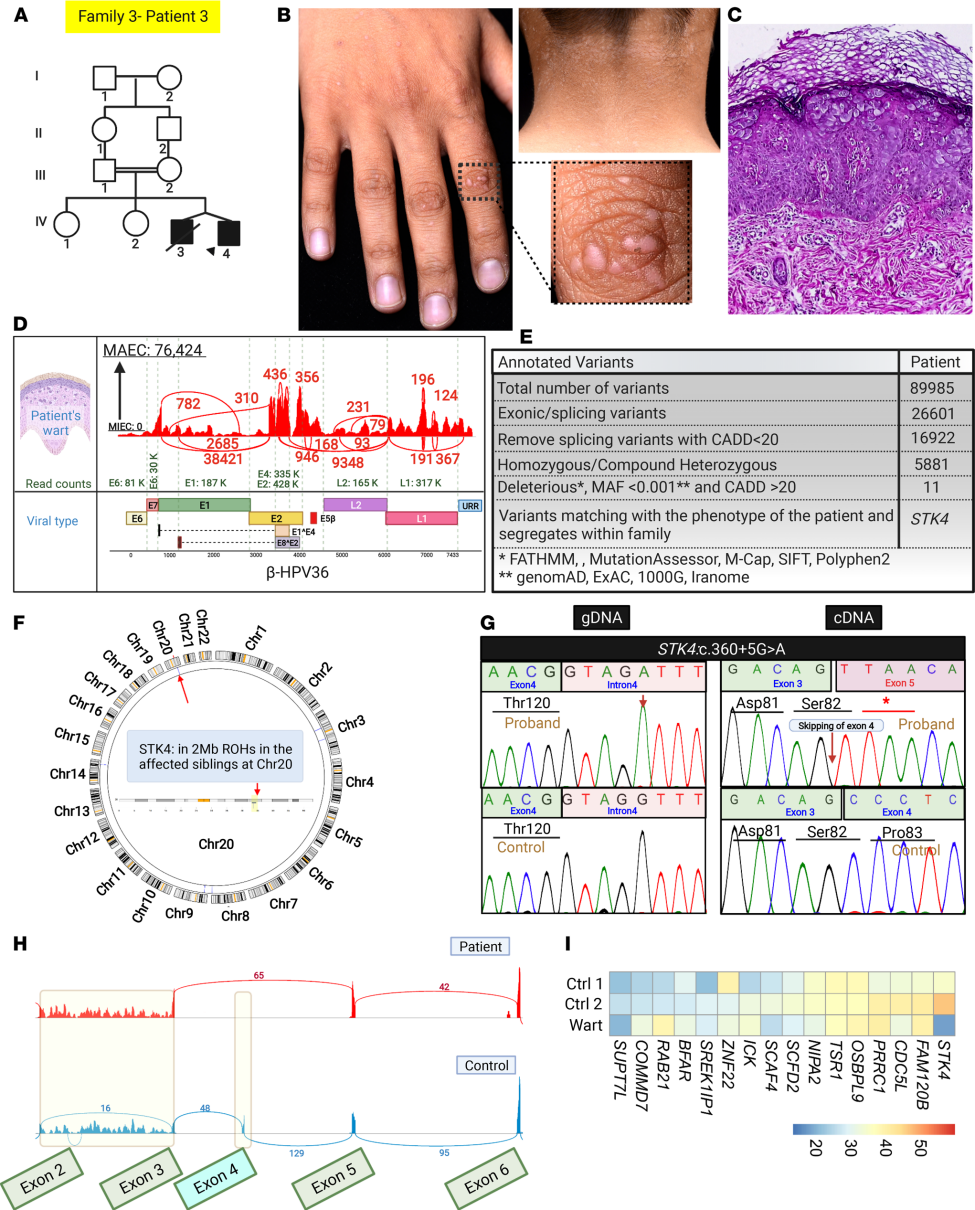
Family pedigree, clinical features, cutaneous histopathology, HPV typing, and identification of *STK4* mutation in a family with IEI. (**A**) Pedigree of family 3, with the proband indicated by an arrowhead, revealed consanguinity. (**B**) Presence of extensive flat warts on the hands and neck of the proband. (**C**) Histopathology of a skin lesion from the proband showed epidermal hyperkeratosis with koilocytes, consistent with HPV infection. (**D**) VirPy detected HPV36 in the wart. (**E** and **F**) Stepwise bioinformatics analyses of variants, along with HM called from RNA-Seq data, revealed *STK4* as the candidate gene. (**G** and **H**) Sanger sequencing and Sashimi plot of the proband’s cDNA and RNA-Seq data confirmed the presence of homozygous splicing variants in *STK4*: c.360+5G>A and the skipping of exon 4. (**I**) Expression of housekeeping and mutated genes in controls versus patient showed downregulation of *STK4*. The figure was generated in BioRender.

In family 3, patient 3, a 12-year-old boy, born in twin pregnancy to healthy first cousin consanguineous parents, presented with the initial diagnosis of EV and extensive recalcitrant warts (Figure 6, A–C). Transcriptome analysis detected HPV36 in his warts (Figure 6D). Transcriptome analysis, assisted by homozygosity mapping, found a noncanonical splice site variant in *STK4*: c.360+5G>A (Figure 6, E and F). HSF prediction, cDNA Sanger sequencing, and Sashimi plot revealed and confirmed the alteration of a wild-type donor site. Therefore, this variant led to skipping of exon 4, initially classified as a variant of unknown significance (VUS) (Figure 6, G–I), and showed a reduction of *STK4* mRNA level.

**Figure 7:**
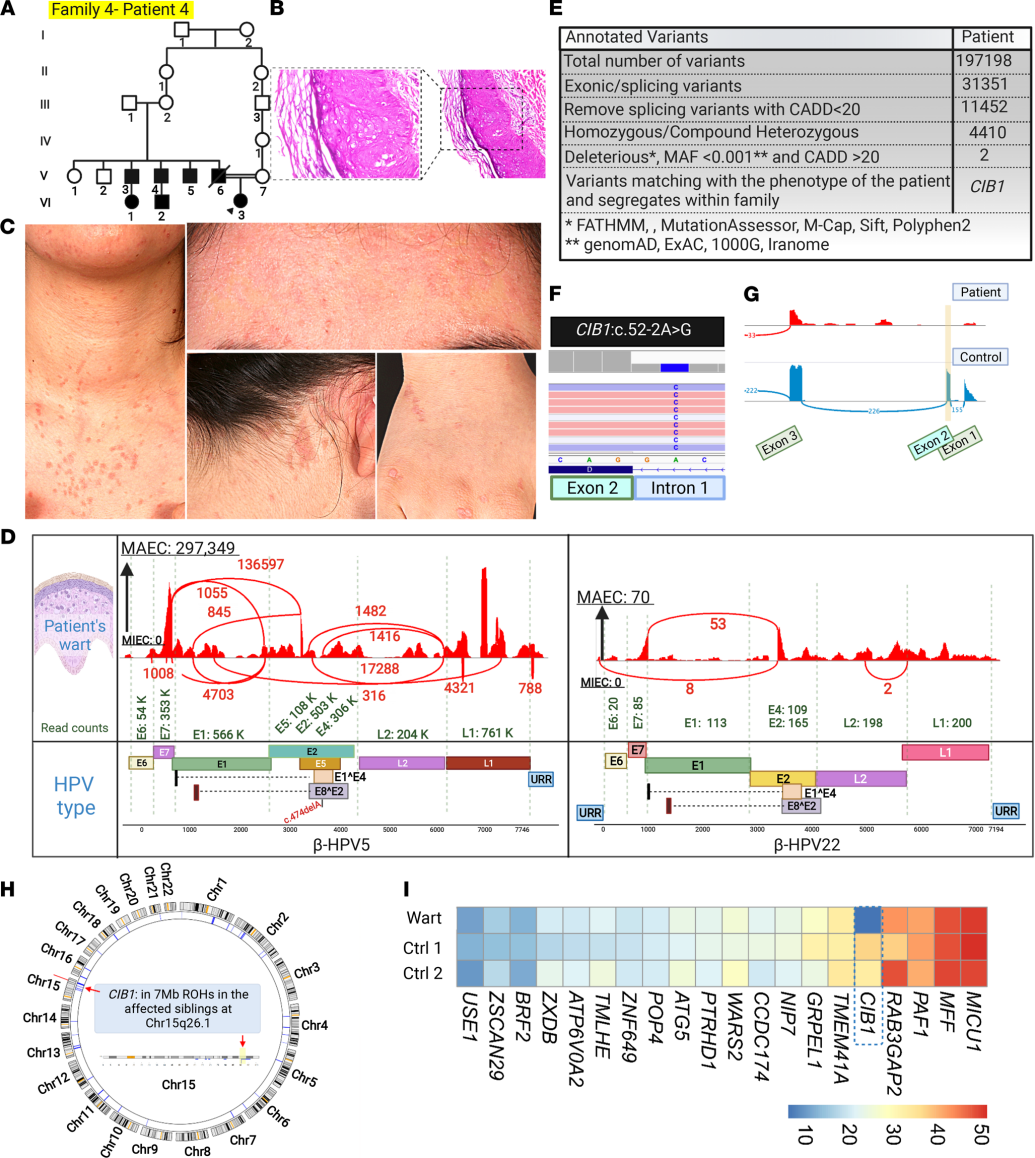
Family pedigree, clinical features, cutaneous histopathology, HPV typing, and identification of *CIB1* mutation in a family with IEI. (**A**) Pedigree of family 4 with the proband indicated by an arrowhead, revealed consanguinity. (**B**) Histopathology of skin lesions from the proband showed epidermal hyperkeratosis with koilocytes, consistent with HPV infection. Original magnification, 10× and 20×. (**C**) Presence of extensive flat warts on the hands, neck, chest, and forehead of the proband. (**D**) VirPy detected HPV5 and HPV22 in the warts of the patient. A sequence variant identified in HPV5 is indicated in red. (**E**–**H**) Stepwise bioinformatics analyses of variants, along with HM called from RNA-Seq data, revealed *CIB1* as the candidate gene. (**G**) Presence of a *CIB1* splice site founder mutation; Sashimi plot confirmed the skipping of exon 2. (**I**) Expression of housekeeping and mutated genes in controls versus patient showed downregulation of *CIB1* mRNA level. The figure was generated in BioRender.

In family 4, patient 4, a 16-year-old girl, born to consanguineous parents, started developing warts at the age of 2. These lesions extend to the hands, neck, and upper chest (Figure 7, A–C). The father had similar lesions, and he developed a squamous cell carcinoma, which metastasized to the brain, leading to his demise at 32 years of age. HPVs 5 and 22 were detected in cutaneous lesions of this patient, HPV5 being the predominant type (Figure 7D). Whole-transcriptome analysis found a canonical splice site mutation in *CIB1*: c.52-2A>G, at the border of intron 1/exon 2, and RNA-Seq results confirmed the skipping of exon 2 (Figure 7, E–I). We recently identified the same mutation in 2 other families with typical EV with different phenotypes and with later onset of skin cancer. By haplotype analysis using SNP markers surrounding the *CIB1* locus (data not shown), we documented the founder effect of this mutation (30).

### HPV detection in patients with IEI with recalcitrant warts.

**Figure 8:**
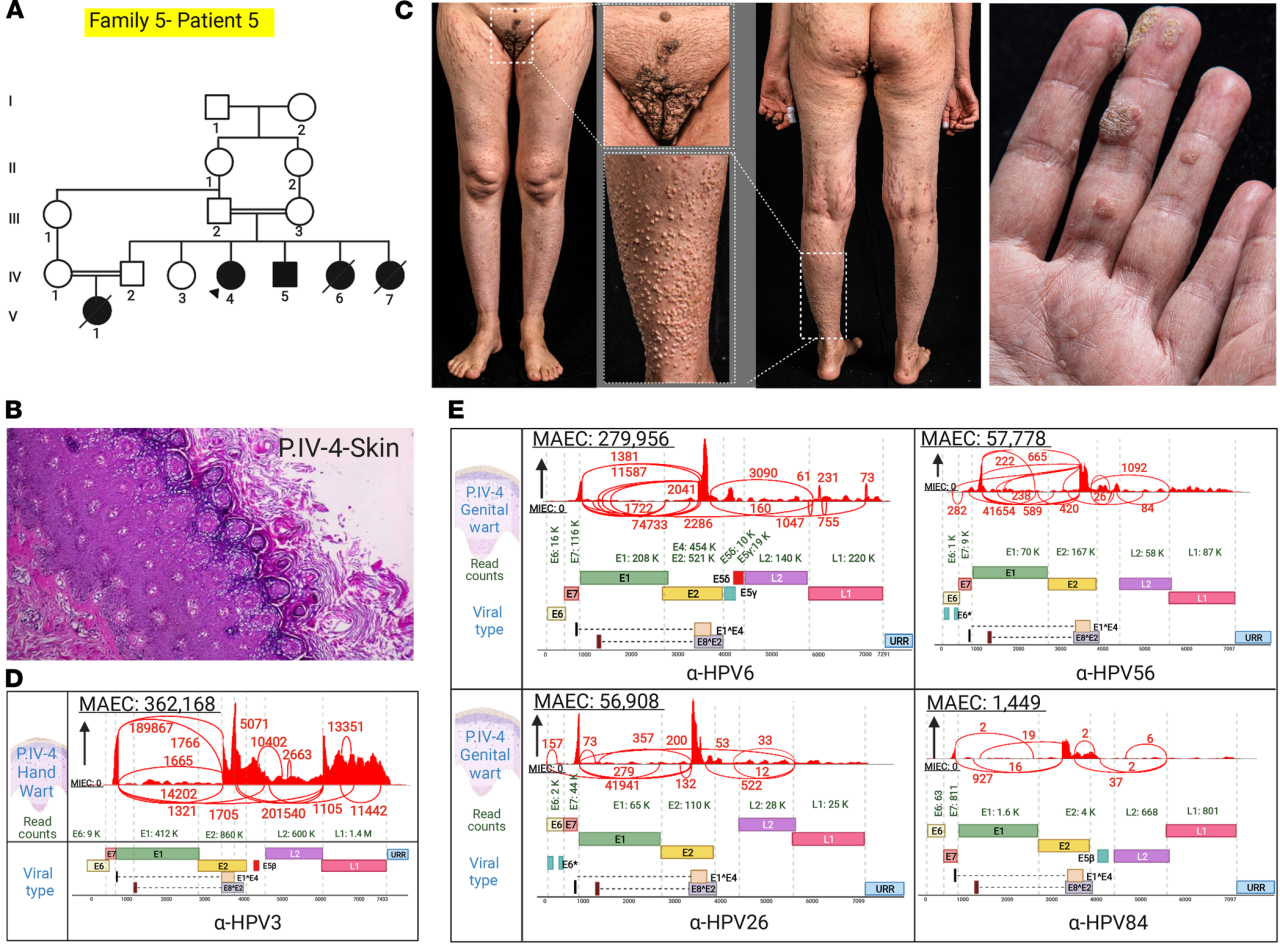
Family pedigree, clinical features, cutaneous histopathology, and HPV typing in a family with *STK4* mutations. (**A**) Pedigree of the family with extensive recalcitrant warts shows consanguinity. The proband is identified by an arrowhead. (**B**) Histopathology of a skin lesion from the proband showed epidermal hyperkeratosis, with keratinocytes showing coarse keratohyalin granules, perinuclear halos, and blue-gray pallor, consistent with HPV infection. (**C**) Widespread distribution of flat and exophytic warts on the genital area, trunk and extremities, as well as palmar hyperkeratosis, in patient IV-4 (patient 5). (**D**) Virome study of patient IV-4 lesions detected HPV3 in warts on the hand. (**E**) We also detected HPVs 6, 56, 26, and 84 in genital warts of patient IV-4 lesions. The figure was generated in BioRender.

**Figure 9:**
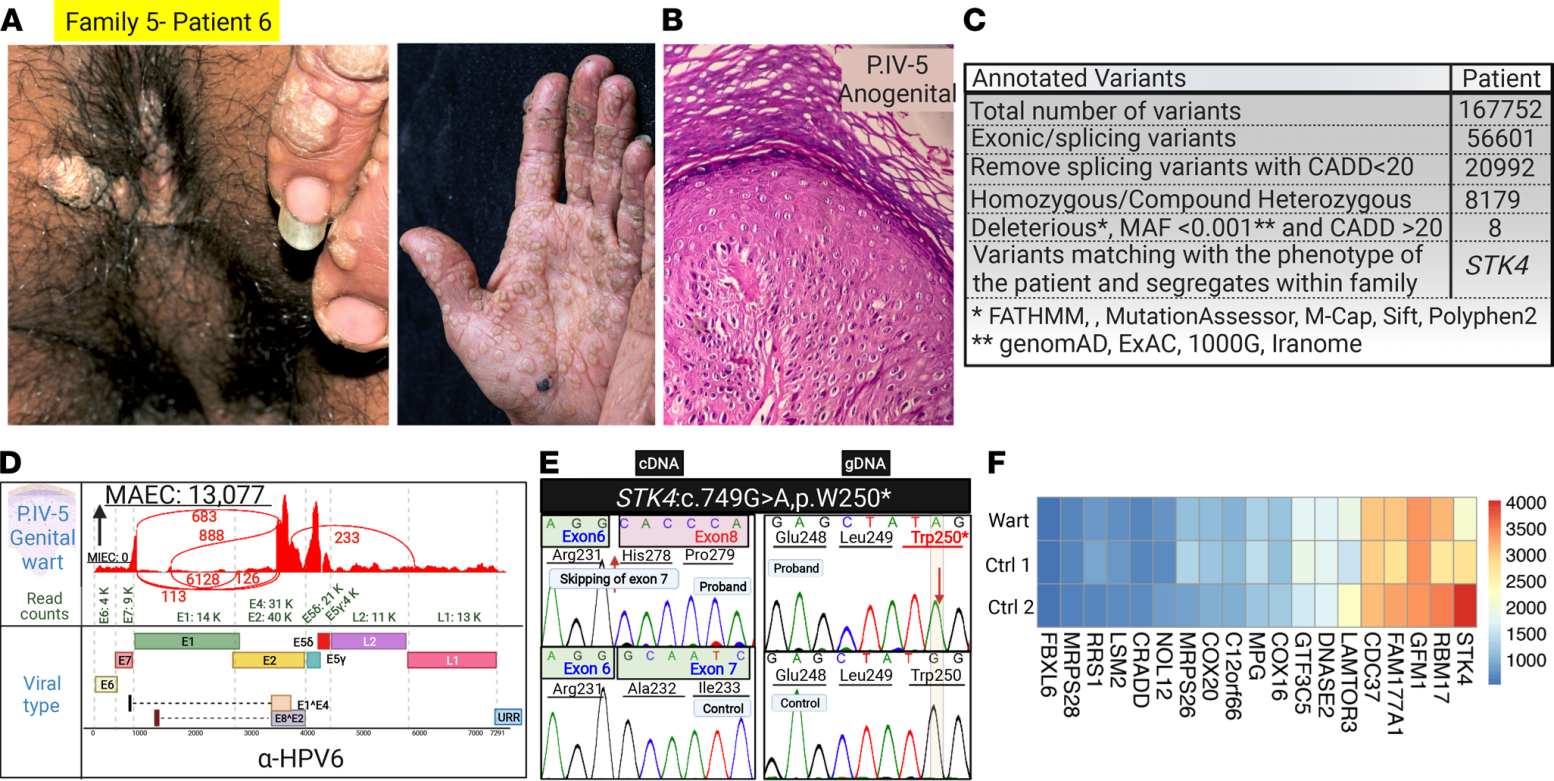
Family pedigree, clinical features, cutaneous histopathology, and HPV typing in the affected sibling in family 5 with *STK4* mutations. (**A**) Anogenital and acral warts in patient IV-5 of family 5. (**B**) Histopathology of anogenital warts in patient IV-5 revealed hyperkeratosis and presence of koilocytes. (**C**) Variant calling using RNA-Seq data followed by bioinformatics filtering identified *STK4* as the candidate gene. (**D**) Virome study of genital warts in patient IV-5 (patient 6) detected HPV6. (**E**) Sanger sequencing of cDNA (left panel) and genomic DNA (right panel) confirmed the presence of the homozygous mutation in *STK4* and the absence of exon 7 in cDNA. (**F**) Heatmap-based expression profiling showed significantly reduced *STK4* mRNA levels compared with randomly selected housekeeping genes, indicating nonsense-mediated mRNA decay. The figure was generated in BioRender. gDNA, genomic DNA.

Family 5, patients 5 and 6 (IV-4 and IV-5), children of consanguineous parents, had extensive cutaneous and genital warts (Figure 8, A–C, and Figure 9, A and B). RNA-Seq was performed on warts in their hands and genital areas. Mutation detection revealed a nonsense mutation in *STK4*: c.G749A, p.Trp250*, and the reverse transcription PCR results showed skipping of exon 7 (Figure 9E). HSF predicted the potential alteration of splicing by alteration of auxiliary sequences and, consequently, substantial alteration of ESE/ESS motifs with ratio of –5.

The warts on the hands of patient 5 contained non-EV HPV3 (Figure 8D). Genital warts of this patient contained HPVs 6, 26, 56, and 84, all in substantial quantities but with HPV6 being the predominant type, as judged by MAEC (Figure 8E). HPV6 was the predominant virus in patient’s 6 genital warts (Figure 9D). Gene expression profiling showed significantly reduced *STK4* mRNA levels compared with randomly selected housekeeping genes, indicating nonsense-mediated mRNA decay (Figure 9F).

**Figure 10:**
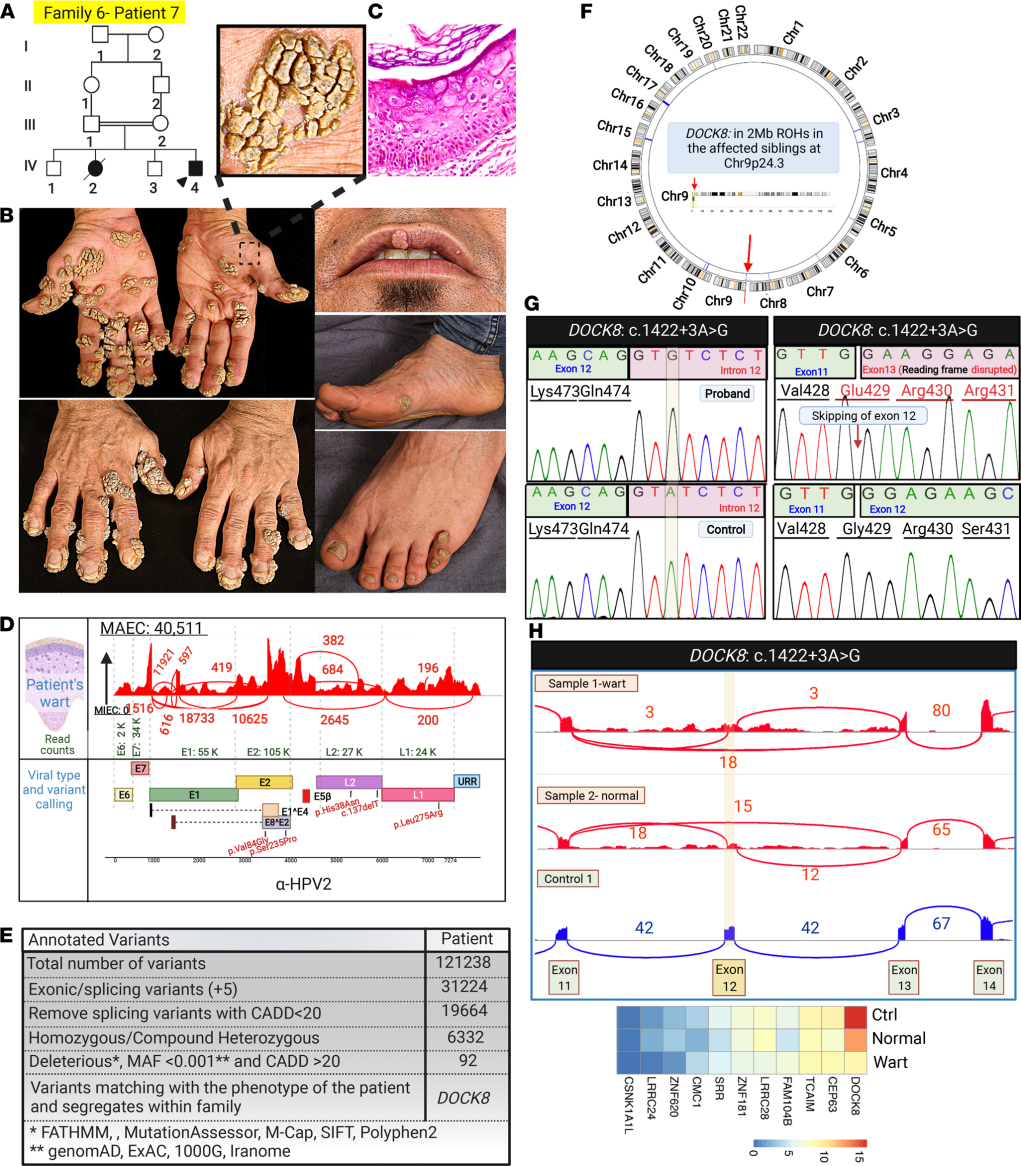
Family pedigree, clinical features, and genetic and viral findings of a family with extensive recalcitrant warts due to IEI. (**A**) Pedigree of family 6 showed consanguinity. The proband is identified by an arrowhead. (**B**) The proband presented with extensive warts throughout the body surface. (**C**) Histopathology of skin lesions showed epidermal hyperplasia with hyperkeratosis and the presence of koilocytes consistent with viral infections. (**D**) VirPy detected the presence of HPV2 in lesional skin. Sequence variants identified in HPV2 are shown in red. (**E** and **F**) Bioinformatics analysis, along with HM from RNA-Seq data, revealed a homozygous splicing mutation *DOCK8*: c.1422+3A>G in family 6. (**G** and **H**) This sequence variant caused partial skipping of exon 12 of *DOCK8*, as shown by cDNA Sanger sequencing and Sashimi plot. (**I**) mRNA expression analysis by heatmap showed downregulation of *DOCK8* transcript compared with a random set of housekeeping genes. The figure was generated in BioRender.

Family 6, patient 7, presenting with extensive cutaneous warts on the extremities, comes from a consanguineous background (Figure 10, A–C). VirPy detected HPV2 and a potentially previously undescribed *DOCK8* c.1422+3A>G variant in this family. This VUS caused partial exon 12 skipping and significantly reduced level of the corresponding mRNA (Figure 10, D–I).

**Figure 11:**
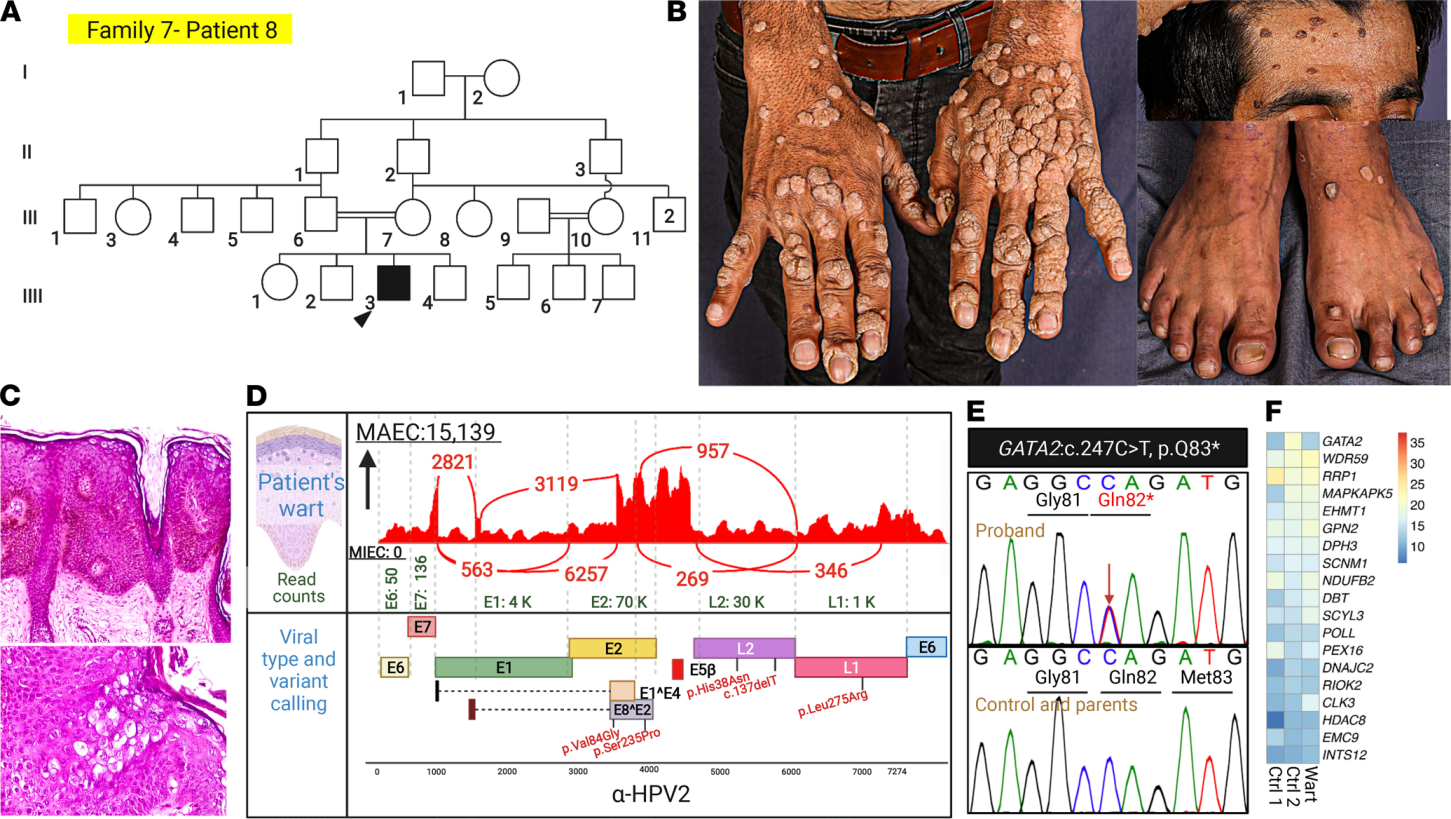
Family pedigree, clinical features, and genetic and viral findings in a family with extensive recalcitrant warts due to IEI. (**A**) The pedigree in family 7 showed consanguinity; the proband is identified by an arrowhead. (**B**) The proband (patient 8) presented with extensive warts. (**C**) Histopathology of skin lesions showed epidermal hyperplasia with hyperkeratosis and the presence of koilocytes consistent with viral infections. (**D**) VirPy detected the presence of HPV2 in a skin lesion. (**E**) Despite the extensive consanguinity, a potentially previously undescribed heterozygous de novo nonsense variant in *GATA2*: c. 247C>T, p.Q83* was found and confirmed by Sanger sequencing. (**F**) mRNA expression analysis by heatmap showed downregulation of *GATA2* transcript compared with a random set of housekeeping genes. The figure was generated in BioRender.

Patient 8 is from the extended consanguineous multiplex family 7. He manifested with cutaneous warts without systemic complications (Figure 11, A–C). The only virus detected in the warts was HPV2, which was absent in biopsies of normal skin from the patient and healthy members of the family (Figure 11D). Despite the consanguineous background, a potentially previously undescribed dominant de novo nonsense mutation in *GATA2*: c.247C>T, p.Q83* was detected in this patient (Figure 11E). mRNA expression analysis by heatmap showed downregulation of *GATA2* transcript compared with a random set of housekeeping genes (Figure 11F).

**Figure 12:**
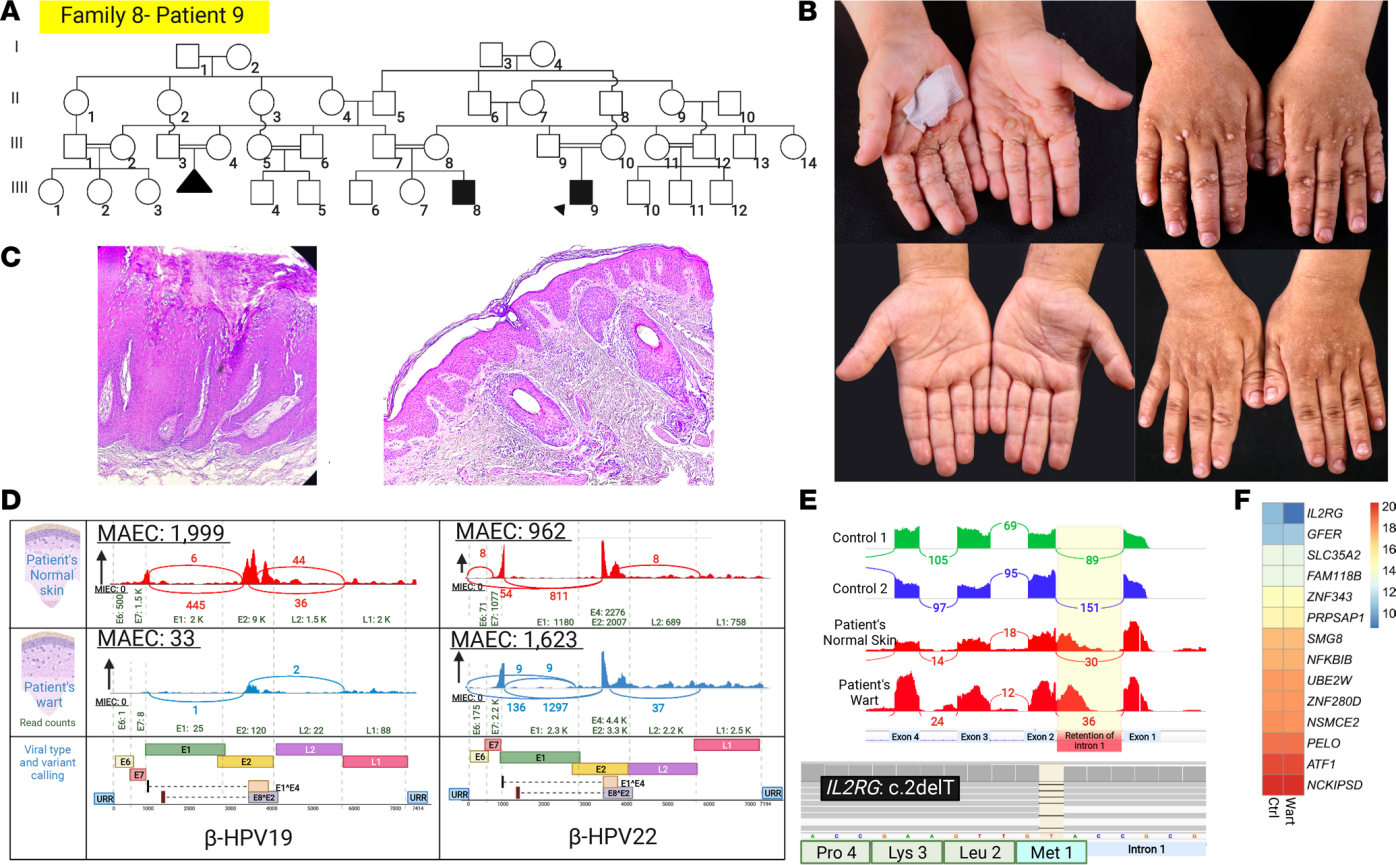
Family pedigree, clinical features, and genetic and viral findings in family 8 with extensive recalcitrant warts due to a mutation in *IL2RG*. (**A**) Pedigree of family 8 with extensive consanguinity. The proband is identified by an arrowhead. (**B**) Extensive recalcitrant warts on the hands (as shown) as well as on the scalp and neck (not shown) of the proband at the age of 4 years (upper panel); these warts spontaneously cleared by the age of 5 years (lower panel). (**C**) Histopathology shows papillomatosis, acanthosis with elongation of rete ridges, mild spongiosis, hypergranulosis, and koilocytosis. There was a sparse lymphocyte infiltration. (**D**) HPV19 and HPV22 were found in both normal appearing skin and a wart. (**E**) A start-loss mutation in *IL2RG* was found, and Sashimi plots showed intron 1 retention. (**F**) Heatmap-based expression profiling showed a significantly reduced *IL2RG* mRNA level compared with randomly selected housekeeping genes. The figure was generated in BioRender.

Family 8, patient 9, has a case of IEI and recalcitrant warts (Figure 12, A–C). HPVs 22 and 19 were detected in both wart and normal skin biopsies at relatively low levels (Figure 12D). The underlying genetic defect was found to be a potentially previously undescribed start-loss mutation in *IL2RG*: c.2delT, p.Met1fs. This variant led to partial intron 1 retention (Figure 12, E and F).

**Figure 13:**
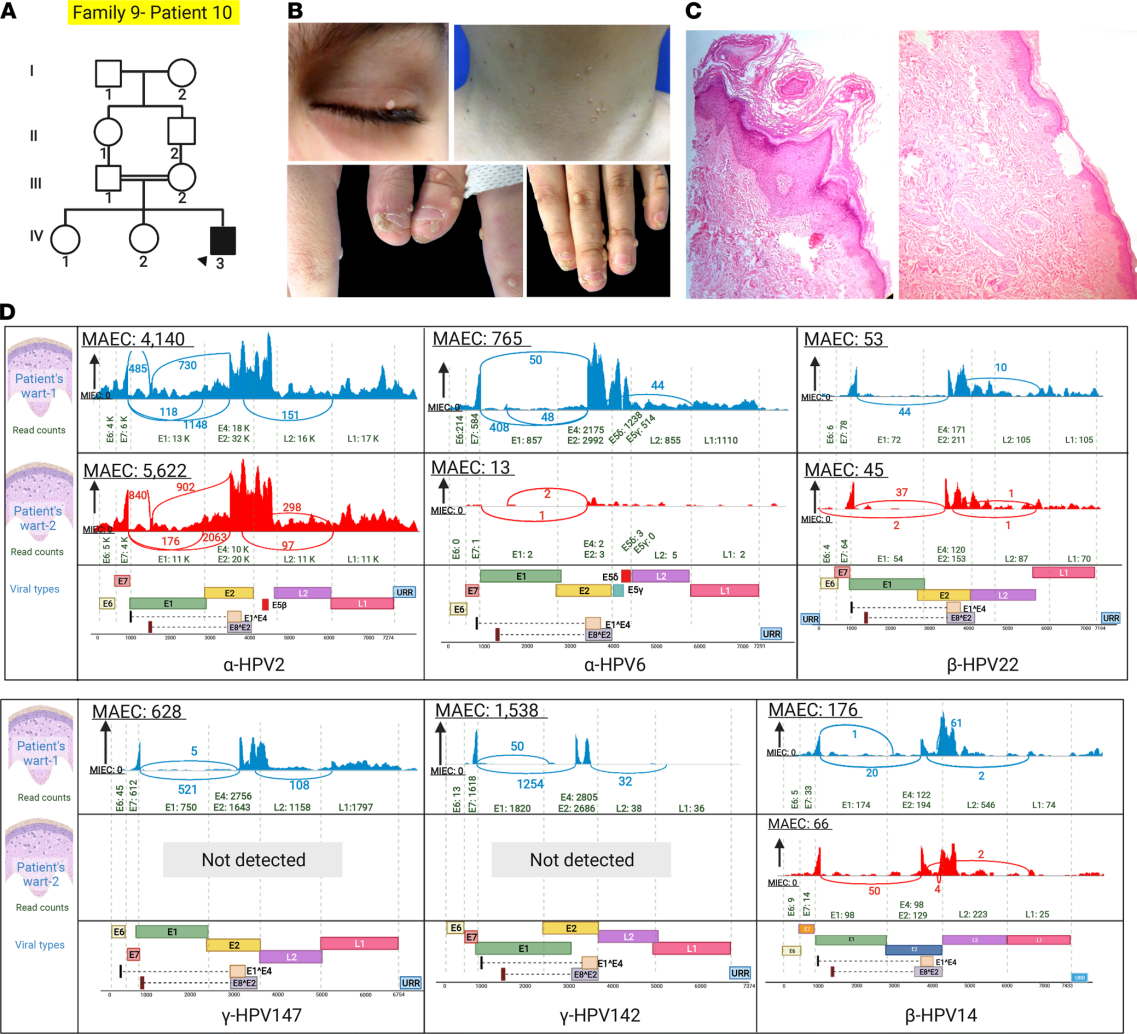
Family pedigree, clinical features, and viral findings in family 9 with extensive recalcitrant warts due to a mutation in *WAS*. (**A**) Pedigree of family 9 with consanguinity. The proband is identified by an arrowhead. (**B**) Extensive recalcitrant warts on the upper eyelid, neck, and hands of the proband. (**C**) Histopathology showed hyperkeratosis with hypergranulosis and irregular acanthosis, and dermal spongiosis is evident. (**D**) HPVs 2, 6, 22, and 14 were detected in both warts while HPV142 and HPV147 were detected only in 1 of the warts of this patient. The figure was generated in BioRender.

**Figure 14:**
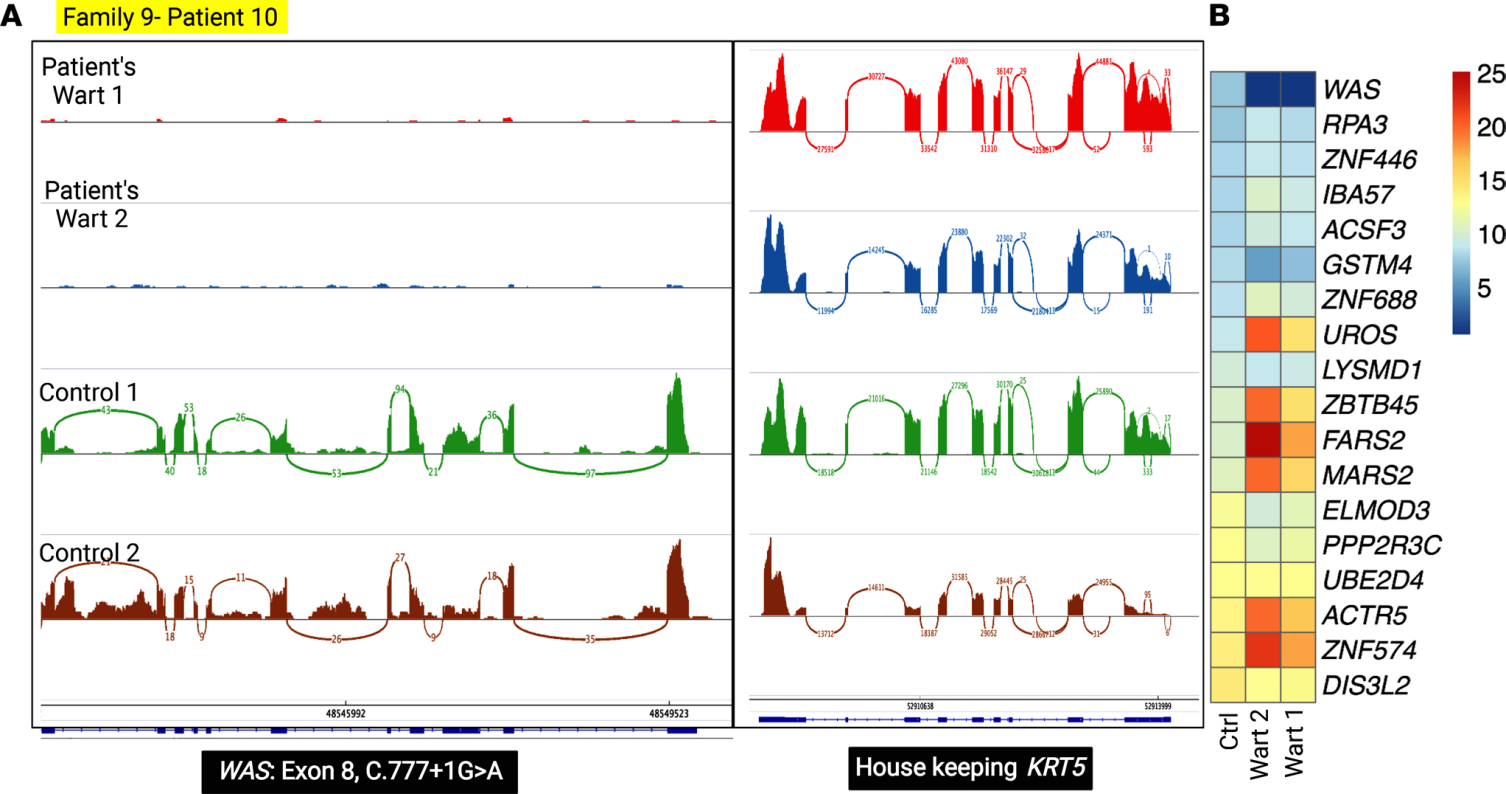
Genetic findings in family 9 with extensive recalcitrant warts due to a mutation in *WAS*. (**A**) A homozygous splicing mutation in *WAS* was found, and Sashimi plots showed complete ablation of the entire *WAS* gene transcript compared with a housekeeping gene. (**B**) Heatmap-based expression profiling confirmed the absence of *WAS* mRNA as compared with randomly selected housekeeping genes. The figure was generated in BioRender.

Another case of IEI, in family 9, patient 10, presented with recalcitrant warts since childhood (Figure 13, A–C). HPVs 2, 6, 22, and 14 were detected in 2 tested warts, with HPV2 being the predominant type, and HPVs 142 and 147 were detected only in 1 wart of this patient (Figure 13D). Mutation analysis revealed a canonical splice site variant *WAS*: c.777+1G>A, and this mutation led to the loss of *WAS* mRNA expression, probably by nonsense-mediated mRNA decay (Figure 14, A and B).

## Discussion

In this study, we propose an automated, web-based application (https://www.VirPy.org) and a standalone pipeline, named VirPy, to identify differentially expressed genes and call variants from RNA-Seq data (bulk and single-cell), simultaneously for both viral and human genomes. Specifically, we integrated a viral detection pipeline from RNA-Seq with our previously published human transcriptome analysis pipeline into an all-in-one method (VirPy) for concomitant analysis of human transcriptome and virome on a biopsy of the lesional and adjacent healthy looking skin, a reservoir for both host epithelial and immune cells. The technique can identify underlying mutations, provide information on the consequences of such mutations, and form the basis for HM in the context of parental consanguinity. To our knowledge, VirPy is the most complete available workflow for analysis of viral species in a single pipeline. VirPy expands upon currently available tools utilizing NGS data to evaluate viral copy numbers, such as VirTect, VirusFinder, Virdetect, HPV-EM, and HPVDetector, complementing these tools with additional downstream analytical techniques for a more complete and robust detection and characterization. When compared with these previously published methods (8–10), VirPy demonstrates improved base functionality of viral detection with added utility for more complete downstream analysis and wider clinical applications.

Several steps in our workflow make improvements to viral detection from similar published workflows, based on the overall computational performance through the implementation of the latest versions of bioinformatics tool packages. Control samples from the healthy appearing skin of the patients, their family members, and unrelated individuals were used to establish the baseline viral expression for each virus detected. In each case, clinically normal skin as a control showed low or absent levels of virus, while in the skin from healthy family members and unrelated controls, no virus was detected. To account for similarities in viral genetic material, such as in closely related types of HPV, the pipeline excludes reads that have primary and secondary alignments to more than 1 viral genome from the quantification step. Interestingly, in several lesions, multiple HPV types were detected, though in most cases one of them was predominant with the highest detected copy numbers.

The VirPy pipeline was also designed to be readily accessible for investigating the virome in clinically derived RNA-Seq data by noncomputational biologists through a web-app-based platform to carry out investigations regardless of access to high-level infrastructure and software knowledge. Implementation of the STAR RNA-Seq aligner with parallelization reduced the total computational time by more than 20-fold compared with methods using single-threaded aligners for human alignment. Secondary alignment of unaligned reads to the viral genome reference was done with HISAT2, as there was no advantage computationally using STAR, with similar output results. Additionally, our pipeline accepts compressed, gun-zipped Fastq files as input, providing users improved flexibility with space efficiency in storing sample data. The multitude of data output files generated for each step in the workflow also allows user flexibility in the analysis using this tool.

Using RNA-Seq as a first-tier diagnostic method has several advantages, such as reclassifications of intronic variants with unknown significance and exonic variants with effect on splicing by alteration of ESS and ESE regions. In particular, in this study, we analyzed the effect of 4 exonic and nonexonic *STK4* variants both by in silico HSF prediction and wet-lab experiments. All of these variants led to aberrant splicing events predicted by HSF algorithms. Reverse transcription PCR and RNA-Seq on patients’ RNA samples confirmed the alteration of normal splicing.

RNA-based, as opposed to DNA-based, NGS is a powerful method to identify virus-induced skin infection, due to the nature and pathogenicity of viruses in human hosts. Viral detection using RNA-Seq captured the expression of active virus mRNA in the skin and provided accurate data for downstream detection and quantification methods. Although DNA-Seq is highly sensitive for the detection of HPV, distinguishing between active infections and the presence of latent, dormant viruses is difficult with this method. RNA-Seq provides high-quality transcription profiles that enable reliable genotyping with subtyping of HPVs and quantification of the viral gene expression. Other advantages of RNA- over DNA-based detection of HPVs include improved specificity, greater predictability of pathogenicity, and precise correlation with histopathology.

Although still new, we believe VirPy has potential clinical utility. For example, our diagnostic pathology center at Thomas Jefferson University reported 1 patient to be HPV negative; however, VirPy was able to detect HPV33 in this patient. The patient was subsequently enrolled in a clinical trial for HPV-positive H&N cancer treatment; without this pipeline, a potentially life-saving intervention may have been delayed.

The primary drawback of VirPy is that it requires a database of known viruses. Although the viral reference database can be expanded upon, the tool cannot detect the presence of new viruses. As of now, we have integrated 926 distinct viruses in the database and will update our database as new viruses are characterized. Another limitation is the lack of detection of retroviral proviruses or endogenous retroviruses and endogenous viral elements using this method. However, the primary focus of this study was only on viruses associated with IEI, especially HPVs. In this study, because patient samples were obtained only from the skin of diagnosed cutaneous lesions, in some cases we were limited in the detecting the expression of viruses that may have low viral load in the normal looking skin.

With expanding application of NGS in clinical settings, there is an increasing demand for bioinformatics tools to process and interpret these generated data. While there are several tools available to perform similar functions in viral detection, there is a paucity of workflows that perform complete, single-run analyses for viral detection, quantification, and visualization as well as concomitant variant calling for causal variants in human genome. VirPy is a complete and robust workflow that addresses this gap by integrating various bioinformatics packages for analysis of RNA-Seq data, and its integration with our previously published pipeline for human transcriptome analysis makes human and virome analysis straightforward from RNA-Seq data.

## Methods

### Study design.

We enrolled patients with cutaneous viral infections with a diagnosis of IEI of unknown genetic etiology (Supplemental Tables 1 and 2). In all cases, the histopathology of the biopsied lesions was consistent with a viral infection. For the documentation and validation of the proof of principle, we also analyzed cases with confirmed presence of HPVs in patients with H&N cancers, the genotypes being confirmed by DNA- and protein-based targeted detection methods, as well as SARS-CoV-2–, HIV-, and EBV-infected cell lines.

### Flow cytometric analysis.

EDTA blood samples were used for immunophenotyping within 12 hours after venipuncture. RBCs were removed using RBC lysis buffer (catalog 00-4333-57, Thermo Fisher Scientific), and lymphocytes were stained with anti-CD3 (document number: IM2705U), -CD4 (document number: IM2468U), -CD8 (document number: IM2638U), -CD16 (document number: IM2642U), -CD19 (document number: IM1285U), -CD20 (document number: IM2638U), -CD27 (document number: IM2578U), and -CD56 (document number: IM2638U) monoclonal antibodies (all from Beckman Coulter). The cells were incubated for 30 minutes, washed twice with PBS, and analyzed by Sysmex CyFlow Space flow cytometer.

### Lymphocyte transformation test.

Peripheral blood mononuclear cells (PBMCs) were isolated from sterile heparinized blood by Ficoll/Hypaque gradient centrifugation. PBMCs were washed twice with RPMI-1640 (catalog 11875093, Thermo Fisher Scientific), counted, and stimulated with phytohemagglutinin (catalog 11249738001, Roche), bacillus Calmette–Guérin, and candida. The negative control contained the cells without any stimulation. The cultures were performed in duplicate and incubated for 6 days in a humidified 37°C incubator with 5% CO_2_. BrdU proliferation assay kit (Roche) was used to measure cell proliferation. The stimulation index was calculated as the mean ratio of the OD of stimulated cells divided by the OD of unstimulated cells.

### RNA-Seq.

RNA-Seq was performed on the RNA extracted from a 3 mm full-thickness biopsy of wart and healthy looking skin. Sequencing was done at the Cancer Genomics and Bioinformatics Core facilities at Thomas Jefferson University using 100 ng of total RNA with the TruSeq Stranded Total RNA Kit (Illumina), with the manufacturer’s suggested protocol. The total mRNA capture and library preparation at 4 nmol/L concentration, including initial barcoding and sequencing on an Illumina NextSeq 500 machine, were performed according to routine procedures at the Thomas Jefferson University genomic facility. With 150 bp paired-end chemistry, a sequencing depth of 50 to 100 million paired reads per sample was achieved.

### Variant calling from RNA-Seq data.

We first used genome mappers, such as BWA or Bowtie2, for alignment of RNA-Seq data to the reference transcriptome (GRCh37/hg19). Next, variant calling of the aligned reads was done by Picard and SAMtools. We identified the final variants through the filtering steps previously described (19), and for families with evidence of consanguinity, overlapping variants with the ROHs were identified.

### Filtration, annotation, and variant prioritization.

We applied the following criteria to enhance the confidence in the accuracy of the called variants: read depth below 10, genotype quality below 20, and variant quality below 50 in vcf files were filtered out.

The variant calling from mapped reads to the reference genome was performed by GATK4 (https://gatk.broadinstitute.org/hc/en-us/articles/360036194592-Getting-started-with-GATK4). We particularly included Filter_reads_with_N_cigar to gain higher variant calling sensitivity. The variant calling region was set to the exons of all transcripts and surrounding 10 bp flanking intronic regions. We used Annovar for determination of the genomic location and functional consequence of called variants (31). Deleteriousness of the variants was predicted using multiple prediction tools, such as FATHMM (http://fathmm.biocompute.org.uk), MutationAssessor (http://mutationassessor.org/r3/), M-Cap (http://bejerano.stanford.edu/mcap/), SIFT (https://sift.bii.a-star.edu.sg), Polyphen2 (http://genetics.bwh.harvard.edu/pph2/), and CADD v 1.4 scores, as shown in Figures 3–8. Variants were checked to be absent from publicly available healthy population databases, such as gnomAD and Bravo. Final variants were identified by superimposing the surviving variants with the ROHs, in families with evidence of consanguinity. Ultimate prioritization of variants employed a 2-level strategy following the latest American College of Medical Genetics and Genomics/Association for Molecular Pathology (ACMG/AMP) and Sherloc classification criteria (32). First, we included variants that were classified by ACMG/AMP as a VUS, as likely pathogenic, and as pathogenic. Second, these variants were refined by Sherloc using a semiquantitative 0 to 5 scoring system. Variants classified as 3-U, VUS, and above were included in the final variant list. The surviving variants were those matched with phenotype and segregate within the family. Final variants were assessed at Genoox Franklin’s website for clinical variant interpretation and annotation (33).

VirPy installation instructions and necessary packages can be found within the GitHub page (https://github.com/Vahidnezhad-Lab/VirPy; commit ID 8e67ee7244e23d21a198180206a5d9dee2570b5f). After installation, the pipeline can be run from command line using “python VirPy.py -1 Fastq1 -2 Fastq2 -o outputDir -index human_reference/ -index_vir viruses_reference/”.

Running the VirPy pipeline creates directories containing measurements of viral quantification, as well as elucidation of viral mutations. The eXpress directory contains the outputs of the viral quantification module, including the main “results.xprs” file. This results file can be opened using a spreadsheet viewer, such as Microsoft Excel. Data elements within the file include viral ID, total mapped read count, unique mapped read count, fragments per kilobase of transcript per million mapped reads, and transcripts per million. Manual analysis of viral quantification and comparison of relative viral abundance within the sample can be done by sorting the unique mapped read count measurement in descending order, with the most abundant viral species appearing at the top of the list. The featureCounts directory contains the outputs of the per virus gene quantification module. Viral gene quantification requires user input of a valid GTF format annotation file per virus. Missing GTF annotation files will be noted for user within the “missingAnnots.txt” file for reference. Each output file within the featureCounts directory contains a unique, delimited text file quantifying gene products per virus detected within the sample. A short list of the detected viruses can be found in the “viruses_detected.txt” file. In addition, the VariantCalling directory contains outputs from the viral mutation analysis module of the VirPy pipeline. An annotated variant call file is created, detailing variants within detected viral species compared to reference genomes obtained from NCBI Virus. Last, viral variants are annotated using SnpEff with predicted effects based on the functional impact on their gene products, found within the “snpEff_genes.txt” file. The final indexed BAM file, which contains the detected viruses, can be visualized, and MAEC can also be obtained from the Sashimi plot feature using IGV. There are regions within viral genome families that are very similar, such that reads may map to multiple viruses. To greatly reduce the risk of detecting nonspecific multimapping alignments, these reads were not counted within the “unique” read count for each virus. Through experimentation, we found that HPV-specific sequence reads below 50 could not be visualized on IGV, and they were difficult to amplify by PCR. By this rationale, we selected 50 as the arbitrary threshold for HPV detection. Also, the unique read count below 50 usually does not consist of enough reads for some HPV genes, such as L or E genes. Therefore, we set 50 as a threshold, but change of this threshold is permissible in different clinical and research settings for clinicians and researchers.

### Validation of VirPy pipeline for detection, quantification, and variant calling on bulk and scRNA-Seq.

A detailed schematic of the steps of the VirPy analysis workflow is shown in Figure 1. Validation and evaluation of the VirPy pipeline were done by analyzing 30 RNA-Seq samples from patients with H&N cancer from the Department of Otolaryngology at Thomas Jefferson University as discussed in detail previously, as well as publicly available data from bulk and scRNA-Seq. The raw FASTQ paired-end reads were obtained from the following experimental systems: human CD56Dim NK cells infected with HIV (Gene Expression Omnibus [GEO] GSE168212) (Figure 2A), HNSCC_PBMC infected with HPV (GEO GSE139324) (Figure 2B), D23_Parenchyma_ROI3_PanCK+ infected with SARS-CoV-2 (GEO GSE162911) (Figure 2C), and primary B cells (EBV-infected) (GEO GSE158275) (Figure 2D), for bulk and scRNA-Seq data.

### Data availability.

Data sets related to this article can be found in National Center for Biotechnology Information’s BioProject with submission identification PRJNA821903 (https://www.ncbi.nlm.nih.gov/bioproject/PRJNA821903).

### Statistics.

*P* values of less than 0.05 were considered significant.

### Study approval.

This study was approved by the Institutional Review Board of the Pasteur Institute of Iran, Tehran, Iran. Written informed consent was obtained from all adult patients and the parents or guardians of children to participate in research and publish their images.

## Author contributions

AHS, LY, VB, EJ, JLC, JU, and HV designed the experiments and prepared the manuscript. AHS, LY, FV, CYH, FP, and HV performed the experiments and the statistical analyses. MN, ZS, HM, AG, S Sotoudeh, MA, NT, AA, SM, MT, SD, S Shokri, MS, NA, SZ, and PF assisted in collecting the samples from patients and healthy controls. FP and NA are responsible for maintenance of GitHub and VirPy websites, respectively, and should be contacted with technical queries. AHS is listed before LY because this study is part of his PhD thesis and he initiated the work.

## Supplementary Material

Supplemental data

Supplemental data set 1

## Version 1 03/22/2022

In-Press Preview

## Version 2 04/22/2022

Electronic publication
