# Supplementary material for: Whole-transcriptome sequencing–based concomitant detection of viral and human genetic determinants of cutaneous lesions
Source: JCI Insight. 2022 Apr 22;7(8):e156021. doi: 10.1172/jci.insight.156021 (PMC9089792; doi:10.1172/jci.insight.156021)
Supplement: Supplemental data [file jciinsight-7-156021-s170.pdf]

Table S1: Demographic and clinical characteristics of patients enrolled in this study.

Patient Code	Sample Types	Sex	Age	Variant	Type of Virus	Unique Viral Counts
Family 1- Patient 1	Non-genital Wart	F	8 Y	*STK4*:c.882delTAATT,P.Leu293*	HPV5	626890
					HPV14	126
					HPV36	87
	Normal Looking Skin				HPV5	13
Family 2- Patient 2	Non-genital Wart	F	16 Y	*STK4:*c.871C>T, p.Arg291*	HPV8	697401
					HPV14	41272
					HPV169	199
	Normal Looking Skin				None	0
Family 3- Patient 3	Non-genital Wart	M	12 Y	*STK4:*c.360+5G>A	HPV36	531422
					HPV130	93
Family 4- Patient 4	Non-genital Wart	F	16 Y	*CIB1:*c.52-2A>G	HPV5	715609
Family 5- Patient 5	Genital-Wart	F	35 Y	*STK4*:c.G749A, p.Trp250*	HPV6	514380
					HPV56	204527
					HPV26	92172
					HPV84	3627
	Non-genital Wart				HPV3	1113946

Family 5- Patient 6	Genital-Wart	M	21 Y		HPV6	42260
					HPV56	664
					HPV26	275
Family 6- Patient 7	Non-genital Wart	M	27 Y	*DOCK8:*c.1422+3A>G	HPV2	200424
Family 7- Patient 8	Non-genital Wart	M	26 Y	*GATA2:*c.247C>T, p.Gln83*	HPV2	91922
Family 8- Patient 9	Normal Looking Skin	M	5 Y	*IL2RG:*c.2delT, p.Met1fs	HPV19	6848
					HPV22	2030
					HPV159	63
					HPV57	56
	Non-genital Wart				HPV19	55
					HPV22	3963
Family 9- Patient 10	Non-genital Wart 1	M	13 Y	*WAS:*c.777+1G>A	HPV2	43294
					HPV6	2965
					HPV147	2451
					HPV142	846
					HPV14	372
					HPV22	225

		HPV17	212
	Non-genital Wart 2	HPV2	30598
		HPV14	174
		HPV22	165
M: Male, F: Female, Y: Year			

Table S2: Immunological assays of the patients with recalcitrant warts indicating primary immunodeficiency

	**Family 1- Patient 1**		**Family 2- Patient 2**			Family 3- Patient 3	Family 5- Patient 5	Family 5- Patient 6	Family 6- Patient 7	Family 7- Patient 8	Family 8- Patient 9	Family 9- Patient 10
Age	6 y	8 y	6 y	11 y	16 y	12 y	34 y	30 y	27 y	26 y	5 y	13 y
Complete Blood Count												
WBC (x*103/dl) (Ref.)	↓2 (4-10)	↓3.6 (5-13)	↓3.7 (4-10)	4.2 (4-10)	↓3.63 (4-10)	↑ 16.95 (4.5-11)	4.93 (4.5-11)	↓ 2.72 (4.5-11)	10.33 (4-11)	5.77 (4-11)	10	↓ 2.65
PMN (x*103/dl) (Ref.)	↓1.2 (3.5-7)	↓2.3 (3.5-7)	↓1.5 (3.5-7)	↓3.1 (3.5-7)	↓2.2 (3.5-7)	↑ 77.9 (40-74%)	58.8 (40-74%)	65.7 (40-74%)	68 (40-74%)	40 (40-74%)	27.7	55%
Lym (x*103/dl) (Ref.)	↓0.7 (2-4.8)	↑7.9 (2-4.8)	↓1.3 (2-4.8)	↑ 9.6 (2-4.8)	↓0.6 (2-4.8)	↓16.4 (20-45)	24 (20-45)	↓16.7 (20-45)	25 (20-45%)	↑ 56 (20-45%)	61.1	35%
Mon (x*103/dl) (Ref.)	0.4 (0.1-1)	↑3.9 (0.1-1)	0.6 (0.1-1)	0.4 (0.1-1)	0.4 (0.1-1)	0.9 (0.2-0.8)	0.79 (0.2-0.8)	↑ 1.09 (0.2-0.8)	5%	2%	7.5	7%
HB (mg/dl) (Ref.)	↓10 (10.7-15.5)	15.6 (11.5-15.5)	13.2 (12-16.4)	↓9.6 (12-16.4)	12.7 (12-16.4)	11.7 (11.5-15.5)	13.7 (13-17.5)	16.7 (13.2-18.5)	14.3 (13-17.5)	↓ 11.1 (13-17.5)	↓ 12.5 (13-17.5)	↓ 11 (13-17.5)
Peripheral Blood Flow Cytometry												

CD3+ (%) (50-85%)	-0.63	1343 (81.6%)	↓(27.3 %)	↓666 (60%)	↓422 (22.4%)	↑81 (60-76%)	83 (58-86%)	↓ 55 (58-86%)	44 (35-78%)	76 (35-78%)	75 (35-78%)	69.5 (35-78%)
CD3+CD4+ (%) (35-60%)	-0.34	669 (49.8%)	↓(18 %)	↓189 (17%)	↓97 (23%)	15 (31-47%)	24 (32-64%)	↓ 16 (32-64)	-	-	-	-
CD3+CD8+ (%) (15-40%)	-	419 (31.2%)	↓(8.5 %)	344 (31%)	↑216 (51.2%)	52 (18-35%)	57 (13-40%)	↑ 41 (13-40)	-	-	-	-
CD3+CD4+CD8+ (%)	-	-	-	-	59 (14%)	0.4	2.3	4.5	↓0.2 (1-3)	0.6 (1-3)	-	0.5
CD3+CD4-CD8- (%)	-	-	-	-	50 (11.8%)	32	4.3	1.31	3	1.7	-	4
CD20+ (%)	-	-	↑(21.6%)	210 (19%)		5.1 (7.1-23.8)	5 (5-22%)	12 (5-22%)	↑ 21.5 (3-15%)	↓ 0.7 (3-15%)	19.8	↓ 1.1
CD19+ (%) (5-20%)	-	122 (7.4%)	↑(20.1)	233 (21%)	203 (10.8%)	5.9 (13-27%)	5 (4.6-21.2%)	11.5 (4.6-21.2%)	↑ 22.5 (3-15%)	1 (3-15%)	19.4	↓ 1.2
CD56+ (%)	-0.09	-	-0.065	67(6%)	-0.045	15.5.1 (3-21%)	6.2 (3-21%)	15.6 (3-21%)	↓1.2 (3-16%)	11 (3-16%)	6.4	↓ 24.5
CD16+ (%)	-0.02	-	-0.122	122(11%)	-0.154	1.4 (6-23%)	6.8 (6-23%)	9.1 (6-23%)	↓1 (3-16%)	14 (3-16%)	8.3	↑ 19.6
CD16+CD56+ (%) (5-30%)		146 (8.9%)	-	-	↑494 (26.2%)	4.7 (6-23%)	4.1 (6-23%)	↓ 4.7 (6-23%)	-	-	-	16

Serum Immunoglobulins												
IgM (Ref.) (mg/dl)	61 (48-207)	↓24 (35-255)	76 (55-210)	67 (55-210)	↑47000 (340-2550)	38.4 (34-214)	65.2 (40-250)	↓ 27.4 (34-214)	↓ 30 (34-214)	↑ 232 (34-214)	59	<39
IgG (Ref.) (mg/dl)	↑2695 (633-1280)	↑1900 (650-1350)	755 (650-1410)	864 (650-1410)	↓980 (6560-13510)	1367.4 (698 - 1560)	1465.4 (700-1600)	1475 (700-1600)	1354 (680-1445)	↑ 1470 (680-1445)	959	1039
IgA (Ref.) (mg/dl)	↑1731 (33-202)	↑1100 (86-320)	255 (83-255)	↑483 (83-255)	950 (860-3200)	↑ 398.1 (58-358)	261.2 (70-400)	↑449.7 (70-400)	369 (83-406)	↑ 3108 (83-406)	94	↓ 34
IgE (Ref.) (IU/ml)	2.7 (<161)	5.9 (<155)	49 (<90)	↑120 (< 90)	↑221 (<200)	↑ 479.6 (<87)	40.1 (<87)	↑803.4 (<87)	↑ 2000 (<175)	45.3 (<175)	↑ 121	2
Serum Autoantibodies												
RF (Ref.)	↑45 (<14)	↑45 (< 14)	-	↑3+	↑3+	↑ 31.1 (0-20)	9.6 (0-20)	18.1 (0-20)	-	-	-	-
ANA (Ref.) <1/80	-	-	-	-	-	-	<1/80	<1/80	0.5	0.5	-	0.2
cANCA (Ref.) <1/20	-	-	-	-	-	<1/20	<1/20	<1/20	-	-	-	-
pANCA (Ref.) <1/20	-	-	-	-	-	<1/20	<1/20	<1/20	-	-	-	-
Lupus anticoagulant (Ref.)	38 (33-41)	40.9 (33-41)	-	-	-	31.2 (33-41)	-	-	35.8 (33-41)	↑ 56 (33-41)	-	37 (33-41)
Anticardiolipin IgM (Ref.) <12	<1 (<10)	3.3 (< 7)	-	-	-	0.16	1.84	0.51	-	-	-	-

Anticardiolipin IgG (Ref.) <12	0.4 (<10)	4 (< 10)	-	-	-	0.66	6.08	0.24	-	-	-	-
Anti-ds-DNA (Ref.) <1/10	2 (<20)	3.3 (< 20)	-	-	-	<1/10	<1/10	<1/10	2.31	2.1	-	2.8
Anti SSA IgG (Ref.) <20	<1 (<20)	↑19.4 (< 15)	-	-	-	1.23	1.63	1.72	3.1	3.6	-	3
Anti SSB IgG (Ref.) <20	3.2 (<20)	7.2 (< 15)	-	-	-	0.36	1.14	1.25	3	3	-	3
Immune Response to Microbes/Vaccines												
Tetanus Ab (Ref.) (IU/ml)	>5 (>5)	3 (>0.5)	-	< 0.1 (≥ 0.5)	0.5 (>0.1)	-	-	-	-	-	-	-
Diphtheria Ab (Ref.) (IU/ml)	>1 (>1)	1.01 (>1)	-	↓< 0.9 (≥ 1)	-	-	-	-	-	-	-	-
CMV IgM (Ref.) (IU/ml)	-	5 (<18)	-	-	-	0.9	0.26	0.26	0.1	0.3	-	0.4
CMV IgG (Ref.) (IU/ml)	-	0.1 (<0.5)	-	-	↑500 (<4)	↑497.03 (<4)	↑ 226.4 (<4)	↑ 153.4 (<4)	↑ 154 (<4)	↑ 88.9 (<4)	-	11.3
EBV IgM (Ref.) (IU/ml)	-	10 (<20)	-	2.7 (< 9)	0.4 (<20)	3.15	2.12	2.14	10	27	-	0.1
EBV IgG (Ref.) (IU/ml)	-	↑748 (<20)	-	2 (< 9)	↑>400 (<25)	↑ 18.34	↑ 29.75	1.85	750	↑ 645	-	10.4
EBV viremia	-	-	-	-	-	-	-	-	-	-	-	-
(↑): Increased value, (↓): Decreased value, (-): Negative results.												

Ref: reference, mg: milligram, dl: deciliter, IU: international unit, ml: milliliter, cm: centimeter
CMV IgM: Negative <0.9, Borderline 0.9--1.1, Positive >1.1
CMV IgG: Negative <9, Borderline 9-11, Positive >11
EBV IgM: Negative <9, Borderline 9-11, Positive >11
EBV IgG: Negative <9, Borderline 9-11, Positive >11
