# Supplementary material for: Whole-transcriptome sequencing–based concomitant detection of viral and human genetic determinants of cutaneous lesions
Source: JCI Insight. 2022 Apr 22;7(8):e156021. doi: 10.1172/jci.insight.156021 (PMC9089792; doi:10.1172/jci.insight.156021)
Supplement: Supplemental data set 1 [file jciinsight-7-156021-s171.pdf]

SUPPLEMENTARY MATERIAL

Case reports:

Family 1: (Figure 4)

The patient was an 8-year-old female born to healthy, first-cousin consanguineous parents with Iranian-Lurish ancestry. After six months of age, she manifested with erythematous papules on her neck, which eventually spread to her forehead, chest, antecubital fossa, and bilateral dorsa of the hands. Skin histopathology demonstrated basket weave hyperkeratosis and papillomatosis of the epidermis. Keratinocytes demonstrated basophilic cytoplasm and perinuclear halo consistent with viral infection. She initially presented with leukopenia and hemolytic anemia at four years of age. Bone marrow histopathology showed hypercellularity with mild dysmyelopoesis and megaloblastic changes, which progressively turned hypocellular (cell-fat ratio 50:50) over three years. She had no history of recurrent skin infections, such as fungal, viral (except HPV) or protozoan, or skin abscesses. Her family history is significant for three siblings who died at an early age due to lower respiratory tract infections. The proband's electrocardiogram and echocardiography did not demonstrate any abnormality.

Family 2: (Figure 5)

The patient was a 16-year-old Iranian-Lurish female born to healthy, double first-cousin consanguineous parents. She initially presented with recurrent and prolonged bacterial/viral upper and lower respiratory tract infections during infancy. She also manifested with suppurative cutaneous abscesses and recurrent inguinal, supraclavicular, axillary, and paratracheal lymphadenopathies throughout the disease course. At four years of age, pale/erythematous non-pruritic macules and papules appeared primarily on the

neck and laterally spread to the chest, face, and upper extremities. Furthermore, she had abdominal pain and bloody diarrhea, which was diagnosed as volvulus and detorted via barium enema at six years of age. Petechiae and purpura appeared on the lower extremities and buttocks several days later. Systemic corticosteroids were administered for three months due to the primary diagnosis of Henoch-Schönlein purpura, and she responded well to the treatment. Chronic abdominal pain and bloody diarrhea recurred at ten years of age. Colonoscopy and rectal mucosal biopsy demonstrated inflammatory bowel disease. Two years later, she manifested with unilateral septic arthritis of the left ankle and right wrist, which was successfully treated with antibiotics. Her family history was insignificant except that the eldest sibling was born at 24 weeks of gestation with a scalp abnormality. The echocardiography revealed mild to moderate tricuspid and pulmonary valve insufficiency. Skin manifestations included reddish, hyperkeratotic lesions on the face, arms, and trunk that developed in early childhood and gradually worsened to form multiple confluent recalcitrant warts. Histopathology of a flat wart in acral skin showed epidermal thickening with hyperkeratosis, acanthosis, and hypergranulosis. Focally, some keratinocytes contained intracytoplasmic eosinophilic inclusions displacing the nuclei.

Family 3: (Figure 6)

The patient was a 12-year-old male, born to consanguineous parents following a twin pregnancy. He began to develop numerous warts on his hands, face, and neck at two years of age. At that time, some abnormalities in liver function tests were found. His twin brother had similar skin findings. The patient died following an episode of jaundice and gastric perforation.

Family 4: (Figure 7)

The patient was a 16-year-old female, born to consanguineous parents, who began developing numerous warts on the hands, head and neck, and upper chest at the age of two years. The number of warts increased as she grew older, and she also developed warts in the genital area. She had no history of squamous cell or basal cell carcinomas. Her father had a similar phenotype, but later developed squamous cell carcinoma on the face at 30 years of age which metastasized to his brain. Despite aggressive treatment, he died two years later.

Family 5: (Figure 8)

Patient 5:

The patient was a 35-year-old female, with past medical history significant for atrial septal defect, multiple episodes of severe bacterial and viral infections that required hospitalization, and non-Hodgkin lymphoma at 31 years of age which was treated by chemotherapy and bone marrow transplantation. She also has multiple recalcitrant warts in different parts of the body including the genital area.

Family 6: (Figure 10)

The patient was a 27-year-old male, born to consanguineous parents, who developed multiple scattered warts by the age of 13 years. Later on, some of warts resolved with the exception of those present on his hands, feet, and lips. He had no other past medical history. He failed multiple treatment attempts, and a skin biopsy was taken for histopathology and HPV typing. Laboratory results showed microcytic anemia with MCV 70.6, Hyper IgE. Level :2000 IU/ml, elevated CD19 and 20, and decreased CD56, 16, and CD3+,

CD4+, and CD8+ cells. He had an older sibling with the same cutaneous findings who passed away at the age of 30 in a motor vehicle accident.

Family 7: (Figure 11)

The patient was a 26-year-old male, born to consanguineous parents. He had multiple episodes of otitis media that resulted in tympanic membrane perforation and sinopulmonary infections that required aggressive medical treatment during the childhood. He started developing numerous warts on the hands, head and neck, upper chest, and back at the age of 14 years. The warts failed to resolve despite several cryotherapy sessions. He did not have history of SCC or BCC.

Family 8: (Figure 12)

The patient was a 5-year-old male who presented with multiple hyperkeratotic, exophytic papules located on his hands. Histologically, there were papillomatosis and acanthosis with elongation of the rete ridges. Koilocytosis was observed in the upper layers of the epidermis. These histopathological features demonstrated common warts. Erythema with mild scale on his face, cheilitis, erythematous patches with excoriations on his neck, and xerosis on the extremities had been present since infancy consistent with atopic dermatitis. Greasy scales adherent to the hairline and vertex region on the scalp appeared during infancy and was confirmed by histopathology to be seborrheic dermatitis which presented for many years. He did not have any history of chronic or severe respiratory infections, gastrointestinal disorders, or opportunistic fungal diseases. His growth was normal, and he had no developmental problems.

Family 9: (Figures 13 and 14)

The patient, a 13-year-old Iranian male, presented with meningitis and persistent thrombocytopenia since the age of 6 months, originally diagnosed as idiopathic thrombocytopenic purpura. At six years of age, he was admitted to the hospital with rectal bleeding and was diagnosed with Crohn disease based on colonoscopy and colonic biopsies. Collectively, his clinical symptoms were consistent with Wiskott-Aldrich syndrome (WAS). The patient were not aware of consanguinity. His mother has multiple sclerosis and his father had polio. The patient was admitted to the hospital with weight loss and bloody stool, and colonoscopy and a colon biopsy confirmed the diagnosis of B cell lymphoma. He received chemotherapy with rituximab, methotrexate, and methylprednisolone. Before starting the chemotherapy, he developed multiple papules on the face and acral extremities that were diagnosed as verruca vulgaris, confirmed by histopathology. The patient reported that the warts started to regress and healed spontaneously.
